# A review on contemporary nanomaterial-based therapeutics for the treatment of diabetic foot ulcers (DFUs) with special reference to the Indian scenario

**DOI:** 10.1039/d1na00859e

**Published:** 2022-04-11

**Authors:** Lakshimipriya Sethuram, John Thomas, Amitava Mukherjee, Natarajan Chandrasekaran

**Affiliations:** Centre for Nanobiotechnology, Vellore Institute of Technology Vellore Tamilnadu India nchandrasekaran@vit.ac.in +91 416 2243092 +91 416 2202624

## Abstract

Diabetes mellitus (DM) is a predominant chronic metabolic syndrome, resulting in various complications and high mortality associated with diabetic foot ulcers (DFUs). Approximately 15–30% of diabetic patients suffer from DFUs, which is expected to increase annually. The major challenges in treating DFUs are associated with wound infections, alterations to inflammatory responses, angiogenesis and lack of extracellular matrix (ECM) components. Furthermore, the lack of targeted therapy and efficient wound dressings for diabetic wounds often results in extended hospitalization and limb amputations. Hence, it is essential to develop and improve DFU-specific therapies. Nanomaterial-based innovative approaches have tremendous potential for preventing and treating wound infections of bacterial origin. They have greater benefits compared to traditional wound dressing approaches. In this approach, the physiochemical features of nanomaterials allow researchers to employ different methods for diabetic wound healing applications. In this review, the status and prevalence of diabetes mellitus (DM) and amputations due to DFUs in India, the pathophysiology of DFUs and their complications are discussed. Additionally, nanomaterial-based approaches such as the use of nanoemulsions, nanoparticles, nanoliposomes and nanofibers for the treatment of DFUs are studied. Besides, emerging therapeutics such as bioengineered skin substitutes and nanomaterial-based innovative approaches such as antibacterial hyperthermia therapy and gene therapy for the treatment of DFUs are highlighted. The present nanomaterial-based techniques provide a strong base for future therapeutic approaches for skin regeneration strategies in the treatment of diabetic wounds.

## Abstract 2

Nanomaterial-based therapeutic treatment shows promising results in enhancing the anti-inflammatory, antimicrobial and angiogenic potential in diabetic rodents for effective wound healing process.

## Graphical abstract

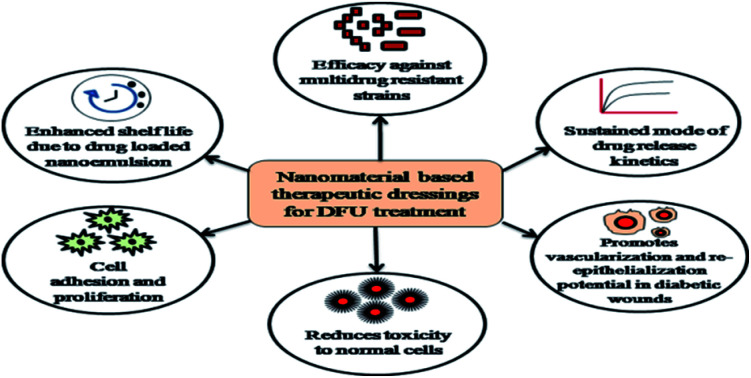

## 1. Introduction

Diabetes mellitus (DM) is a global syndrome characterized by an excessive hyperglycemic state. According to the International Diabetes Federation (IDF), approximately 536 million (20–79 years) adults are living with diabetes as of 2022 in India, and this number is projected to rise to 645 million by 2030 and 784 million by 2045.^1,2^ There are many diabetes-related complications, such as diabetic ketoacidosis, nerve damage, hypoglycemia, mastopathy, kidney-related diseases, cardiovascular diseases, necrobiosis, retinopathy, hyperosmolar acidosis, and musculoskeletal conditions; however, the most prevalent is diabetic foot ulcers (DFUs).^3,4^ Among the affected diabetic individuals worldwide, 20% of patients develop diabetic wounds (DWs).^5^ A diabetic wound/foot ulcer infection is a critical complication in diabetic patients, which takes time to heal, resulting in the degradation of skin tissues and exposure of cellular layers.^6,7^ Specifically, non-healing (chronic) DFUs are associated with complications such as foot deformity, wound infections, and finally limb amputation.^8^ The strategies for the treatment of DWs include tissue debridement, stem cell-based therapies, hyperbaric oxygen therapy, negative pressure wound therapy, photobiomodulation therapy, antimicrobial photodynamic therapy, vacuum-assisted closure therapy, ultrasound-mediated therapy and revascularization therapy to retain the blood flow. These treatment options are beneficial for wound closure and wound contraction, but understanding the wound pathophysiology is a challenging task, resulting in prolonged healing time and wound recurrence, leading to limb amputations.^9–12^ The major limitation of antibiotic therapy and wound dressings in chronic wounds is the inadequate and improper supply of antibiotics and therapeutics to the target cells. This phenomenon tends to decrease the impact of medications and results in antimicrobial resistance (AMR). Thus, drug-targeted delivery is essential for treating both acute and chronic wounds.

The poly or monomicrobial nature of chronic wound infections is characterized by the formation of biofilms, leading to AMR due to the poor permeability of the biofilm matrix. With the increasing percentage of biofilm infections, it is necessary to develop non-commercial antimicrobial treatments, such as nanomaterials that possess intrinsic anti-biofilm properties by modulating their biophysical or biochemical parameters to cause removal and disruption of biofilms, such as synthesizing nanomaterials as drug delivery paradigms for carrying bioactive compounds, antibiotics, antioxidants, growth factors and stem cells to infection sites for better incursion through the biofilm matrix.^13^ Nanomaterials can be used to treat chronic foot ulcers because they help in modulating biofilm formation and microbial colonization in wounds based on their different particle shapes, compositions, sizes and surface charges, resulting in alterations in the composition of the bacterial cell membrane and generation of reactive oxygen species (ROS), lipid peroxidation, loss of respiratory activity, nitrosation of cysteines and DNA unwinding of metabolic pathways.^14^ Due to the heterogeneity of nanomaterials, they can serve as effective platforms to deliver anti-inflammatory, anti-biofilm and angiogenic properties based on the pathophysiological condition of the wound site. The nature, state, depth, exudates, healing pace and comorbidities of the wound suggest the appropriate nanoplatform to be applied for infection control, which can possibly change the milieu from non-healing to healing.^15^ Some the suitable nanomaterial-based platforms such as organic platforms (nanoemulsions, nano hydrogels, nanoliposomes, and nanofibers) and inorganic platforms (metallic and non-metallic nanoparticles) have given a new dimension towards chronic wound healing treatment strategies. In the current scenario, nanomaterial-based diabetic wound healing approaches act as powerful weapons against multi-drug-resistant infections and transdermal nanocarriers and possess intrinsic regenerative properties, nanoscaffolds, and nanotopography to prevent biofilm formation, providing cell-type specificity benefits unlike the conventional wound dressings or available therapies.^15–17^

Teaima *et al.* fabricated polyurethane-modified chitosan nanofibers encapsulated with various concentrations of linezolid in a diabetic experimental model. The results illustrated that linezolid promoted diabetic wound healing and control the microbial growth at the wound site. This type of strategy plays an important role in the treatment of acute and chronic wounds. The wound healing can be enhanced by the capability to infuse growth factors and epidermal cells. Thus, the nanofibers were loaded with various concentrations of linezolid, which exhibited a fast release step associated by slow and more steady release. The percentage of wound contraction for the treated groups (linezolid-loaded nanofibers) was higher compared to the control groups.^18^ Tallapaneni *et al.* determined the effects of resveratrol microparticles encapsulated with chitosan-collagen scaffold-associated doxycycline (RES-DOX-CS-CLG) for the treatment of diabetic wound healing. The RES-DOX-CS-CLG scaffold was found to be biocompatible and resulted in enhancing cell proliferation and development compared to the control groups. The ability of the drug-loaded DOX-CS-CLG scaffold to promote wound closure and the effects of the migratory capability of 3T3 fibroblast cells were investigated. After 24 h, the cells treated with the scaffold migrated much quicker than the control samples.^19^ Ren *et al.* fabricated anti-inflammatory and antibacterial Ag@hesperidin core–shell nanoparticles embedded in nanofibers for the treatment of an infected wound. These nanoparticles presented effective antibacterial properties against *E. coli* and *S. aureus*. The Ag–Hes NPs exhibited a high scavenging ability of 69%. Under the influence of sodium alginate and polyvinyl alcohol, the Ag–Hes NPs were loaded in electrospun nanofibers to form a hydrogel. Ag–Hes@H promoted the proliferation and migration of endothelial cells, and thereby resulted in accelerated infected wound healing. Thus, the designed anti-inflammatory nanomaterials possess great potential for chronic wound healing applications.^20^ Nanomaterials have the ability to eradicate multi-resistant bacteria in the biofilm matrix. Additionally, drug-loaded nanomaterials play an essential role in targeting bacterial cells and act as efficient drug delivery paradigms for chronic infected wound healing applications. Therefore, nanomaterials can be an excellent toolkit for the development of various treatment strategies using different nanoplatforms against multi-drug resistant (MDR) biofilm and planktonic infections.^21^ Accordingly, there is an urgent need to discuss the various nanomaterial-based therapies available for preventing AMR and biofilm-related chronic infections, delaying wound healing and ischemic (poor blood flow) disorders, and ultimately preventing limb amputations.

In the present review, the status and prevalence of DM and diabetic wound amputations in India are highlighted. In addition, the pathophysiology, complications and current therapies of DFUs are discussed. The recently emerging line of DFU treatment using nanomaterials such as nanoemulsions, nanoparticles, nanofibers and nanoliposomes is discussed in this review. In addition, nanomaterial-based innovative therapies such as antibacterial hyperthermia therapy and gene therapy for DFUs are studied.

## 2. The status and prevalence of diabetes mellitus (DM) and diabetic wound amputations in India

**Fig. 1:**
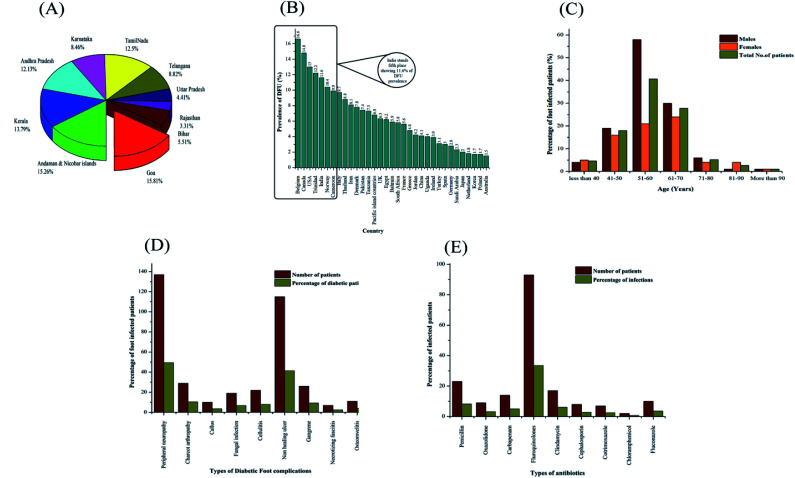
(A) Prevalence of diabetes mellitus (DM) in India. (B) Country-wise prevalence of diabetic foot ulcers (DFU). (C) Prevalence of foot-infected diabetic patients depending on age. (D) Prevalence of diabetic foot complications among foot-infected diabetic patients. (E) Various types of antibiotics provided for the treatment of diabetic foot ulcer (DFU) patients.

India has become the capital of diabetes in the world, which affects millions of its population. India is ranked second after China, where there are more than 66.8 million diabetics in the age group of 20–70 years. According to Yadav *et al.*,^22^ the prevalence of DM is expected to increase gradually to 370 million by 2030 with a rapid and faster rise in India followed by China with 42.3 million and United States of America with 30.3 million. According to the National Diabetes and Diabetic Retinopathy Survey published by the Ministry of Health and Family Welfare, the prevalence of DM was 12.2% in individuals over the age of 50, while that in individuals under the age of 50 is 7.5%, and the percentage of prediabetic individuals is 5.7%. In the case of diabetic retinopathy, 16.9% of individuals over the age of 50 years was found to be affected, 18.6% of individuals in the age group of 60–69 years affected, 18.3% of individuals in the age group of 70–79 years affected and 18.4% of people above the age of 80 years severely affected with eyesight problems.^23^ The Government of India has recently initiated the National Program for Diabetes and Cardiovascular Diseases to set up camps for the screening and diagnosing diabetes-related symptoms. Fig. 1(A) shows the prevalence of diabetes mellitus (DM) in India.

As seen in Fig. 1(A), Goa has the highest prevalence of DM with 8.6%, followed by Andaman and Nicobar Islands and Kerala with 8.3% and 7.5%, respectively. Among the states, Goa has the highest diabetes prevalence among men, while Kerala shows the highest diabetes prevalence among women. The diabetes prevalence in Uttar Pradesh, Arunachal Pradesh, Assam and Rajasthan was below 5% among women, while only Mizoram and Rajasthan had a diabetes prevalence level of 5% among men. The economically more prosperous states (*e.g.*, Goa and Kerala) are expected to exhibit higher rates of diabetes compared with the other states (*e.g.*, Rajasthan), which is specifically mediated by higher calorific diets and much lower levels of physical exercise.^24–26^ Preliminary observations from a survey reported by the Indian Council of Medical Research (ICMR) revealed that Jharkhand and Chandigarh have 0.96 and 0.12 million DM cases, compared to that of Maharashtra and Tamilnadu, showing 9.2 and 4.9 million, respectively. The national survey reported in various metropolitan cities suggests similar trends, *i.e.*, 11.8% in Kolkata, 11.6% in New Delhi, 9.3% in West India and 6.1% in Kashmir Valley compared to Hyderabad, Chennai and Bangalore, which showed 16.6%, 13.5% and 12.4%, respectively.^27,28^ The level of mortality and morbidity due to the incidence of DM is alarming and poses a burden on the healthcare in both society and family. This chronic disease is prevalent across India, posing significant demands for urgent research interventions at the national and regional levels to mitigate the catastrophic acceleration in DM predicted for upcoming years.

DFUs are one of the greatest issues of DM, which together other serious complications cost nearly 1960 USD to treat. Consequently, patients in India need 5.7 years of income to access DFU treatment. In India, although the present population-based report is not available, it is observed that approximately 45 000 legs are amputated annually in India.^29,30^ More than half of DFU patients become highly infected, which requires prolonged hospitalization, while 20% of them result in amputations. After amputation, 60% of DFU patients have their other limb amputated within the next two years. However, the management of DFUs in India relies totally on the use of neuropathic medications and antibiotics.^31,32^ Compared to other middle income countries, the type of treatment for DFUs in India includes neuropathic drugs, growth factors, wound therapy, dressings, collagen scaffolds, negative pressure wound therapy and incisional/excisional surgery. Some rural areas in India suffer from a lack of education and poverty, leading to inappropriate footwear and severe foot lesions. This problem is exacerbated by the extended delay in accessing healthcare providers because patients tend to approach alternative medical prescribers and informal healthcare providers. The cost of DFU treatment in India has been found to be nearly 19599/patient (USD).^29,33,34^

A statistical survey on the country-wise prevalence of diabetic foot ulcers is shown in Fig. 1(B). According to statistics, Belgium, Canada, USA, Trinidad, India, and Norway have a reported percentage of prevalence higher than 10. Countries such as Greece, Jordan, China, Uganda, Ireland, Turkey, Spain, Germany, Saudi Arabia, Japan, Netherland, Korea, Poland and Australia have a reported lower prevalence of diabetic foot ulcers, as shown in Fig. 1(B). A meta statistical analysis elucidated the geographical variance in DFU prevalence, where Belgium shows the highest percentage of DFUs with 16% and the lowest prevalence of DFUs was found in Australia with 1.2%. The report evaluated nearly 67 studies with various types of methodologies in the health sector unit. Most studies were reported in Europe and Asia and covered approximately 801 985 participants. Various risk factors have been reported including type II DM, body mass index, age, and gender, and many other complications such as peripheral vascular disease and diabetic nephropathy have been identified.^35,36^

DM may also result in the dysfunction of organ systems, presenting as immune, nervous and expand integumentary disorders, while DFU is associated with severe pain, extended hospitalization, multi-treatment regimens, increased mortality and decreased mobility rate. Approximately 15–30% of diabetic individuals attain DFUs, leading to substantial wound management throughout their life.^37,38^ The incidence of diabetes accounts for 8 out of 10 nontraumatic-type amputations. The percentage mortality ranges from 15–42% in 2 years, 34–64% in 4 years and 40–80% in 6 years.^39^ Among the DFU patients, the majority of foot ulcers are neuropathic, 18.7% ischemic, and 34.2% neuroischemic. Approximately 3% of DFU patients result in amputations. Wound infection is considered as the major reason for amputation in 90% of diabetic patients.^40^ The incidence of DFUs is increasing at an alarming rate worldwide. The incidence of DFUs also depends on age and gender. In India, during a particular period, 4.6% (no. of males: 4 and no. of females: 5) of diabetic patients are under the age of 40 years, 18% (no. of males: 19 and no. of females: 16) of diabetic patients between the age of 41 to 50 years, 40.7% (no. of males: 58 and no. of females: 21) of diabetic patients between the age of 51 to 60 years, 27.8% (no. of males: 30 and no. of females: 24) of diabetic patients between the age of 61 to 70 years, 5.2% (no. of males: 6 and no. of females: 4) of diabetic patients between the age of 71 to 80 years, 2.6% (no. of males: 1 and no. of females: 4) of diabetic patients between the age of 81 to 90 years, 1% (no. of males: 1 and no. of females: 1) of diabetic patients above the age of 90 years are affected with serious diabetic foot infections, as shown in Fig. 1(C). These statistics show that diabetic patients between the age of 51 to 60 years (approximately 40.7% of DFU patients) are affected severely with diabetic foot infections due to high calorific diet intakes and improper health check-ups, leading to higher rates of limb amputations.

There are various diabetic foot complications associated with diabetic patients such as peripheral neuropathy, Charcot arthropathy, callus, fungal infection, cellulitis, non-healing ulcer, gangrene, necrotizing fasciitis and osteomyelitis. In India, on an average a month, the number of patients affected with peripheral neuropathy was found to be 137 and the percentage was nearly 49.45%, 29 patients (10.46%) affected with Charcot arthropathy, 10 patients (3.61%) affected with callus, 19 patients (6.85%) affected with fungal infections, 22 patients (7.94%) affected with cellulitis, 115 patients (41.51%) affected with non-healing ulcers, 26 patients (9.38%) affected with gangrene, 7 patients (2.52%) affected with necrotizing fasciitis and 11 patients (3.97%) affected with osteomyelitis, as depicted in Fig. 1(D). The number of DFU patients affected with peripheral neuropathy (137 patients) and non-healing ulcers (115 patients) is increasing at a faster rate compared with the other foot complications such as Charcot arthropathy, callus, fungal infections, cellulitis, gangrene, necrotizing fasciitis and osteomyelitis. Different types of antibiotics such as penicillin, oxazolidone, carbapenem, fluoroquinolones, clindamycin, cephalosporin, cotrimoxazole, chloramphenicol and fluconazole are used for the treatment of DFUs. In a pediatric clinic in South India, there were 23 patients on average (8.3% of diabetic patients) treated with penicillin, 9 patients (3.2% of diabetic individuals) treated with oxazolidone, 14 patients (5.05% of diabetic individuals) treated with carbapenem, 93 patients (33.5% of diabetic individuals) treated with fluoroquinolones, 17 patients (6.1% of diabetic individuals) treated with clindamycin, 8 patients (2.8% of diabetic individuals) treated with cephalosporin, 7 patients (2.5% of diabetic individuals) treated with cotrimoxazole, 2 patients (0.7% of diabetic individuals) treated with chloramphenicol and 10 patients (3.6% of diabetic individuals) treated with fluconazole, as shown in Fig. 1(E). According to the above-mentioned observations, it can be concluded that fluoroquinolones are given frequently for the treatment of DFUs. However, in some cases, the treatment of DFU patients with antibiotics leads to multi-drug resistant infections such as methicillin-resistant *Staphylococcus aureus* (MRSA) and vancomycin-resistant *Staphylococcus aureus* (VRSA).

Recently, it has been reported that a drug-coated stent was used for angioplasty by a team of doctors at Mumbai's Symbiosis Speciality Hospital, which is considered a medical breakthrough for DFU treatment in India, in a 54 year-old female patient who suffers from a longstanding history of uncontrolled DM and foot ulcers to save her limbs from amputation. The patient developed a small blister on the third toe after an accidental injury. The arterial pulses in the foot were found to be weak on manual palpation. The examination by the doctors showed poor blood flow in the legs due to uncontrolled diabetes, which could necessitate major limb amputation. After examining the level of the blood flow blockage, a lower limb angiogram procedure followed by femoral artery angioplasty and stenting at the level of blockage was planned. This latest drug-eluting femoral stent was found to be biocompatible in treating non-healing foot ulcers (Hindustan Times, 2022). Alkem Laboratories Ltd. (Alkem) announced their launch of unique and patented 4D bioprinting technology in the latter half of 2022, post-regulatory approval for treating non-healing chronic and deep wounds. This technology is expected to be available at affordable rates to DFU patients and will exhibit high scope for preventing limb amputations (Financial Express, 2022).

Although there are very few findings on the treatment of non-healing chronic foot ulcers, researchers should mainly focus on the latest targeted delivery-based treatments and strategies related to nanotechnology, which can find a way to prevent limb amputations and expand novel therapeutic tools for the treatment of DFUs.

## 3. Pathophysiology of diabetic foot ulcers (DFUs)

**Fig. 2:**
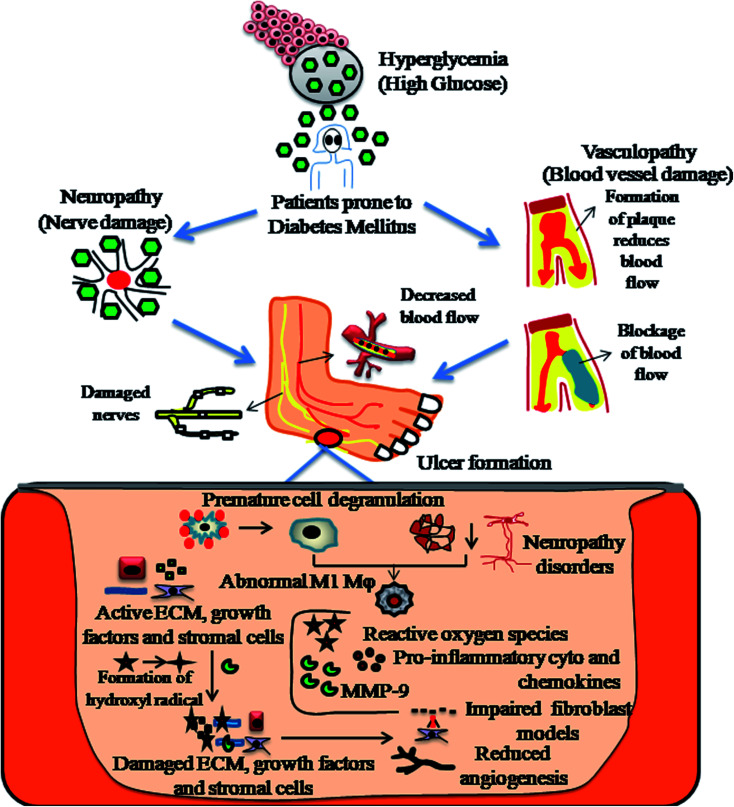
Diagrammatic representation of the pathophysiology of diabetic foot ulcers (DFU).

Diabetic wound healing necessitates synergy between biochemical mediators and inflammatory cells, stimulated by different factors. Monocytes transformed into cellular macrophages are considered to be the predominant producers of various pro-inflammatory cytokines, namely, IL-1β, IL-6, IGF-1, TGF-β, TNF-α and VEGF, involved in both normal wound healing and diabetic healing processes.^41,42^ The detailed pathophysiology of DFU is explained in Fig. 2.

**Fig. 3:**
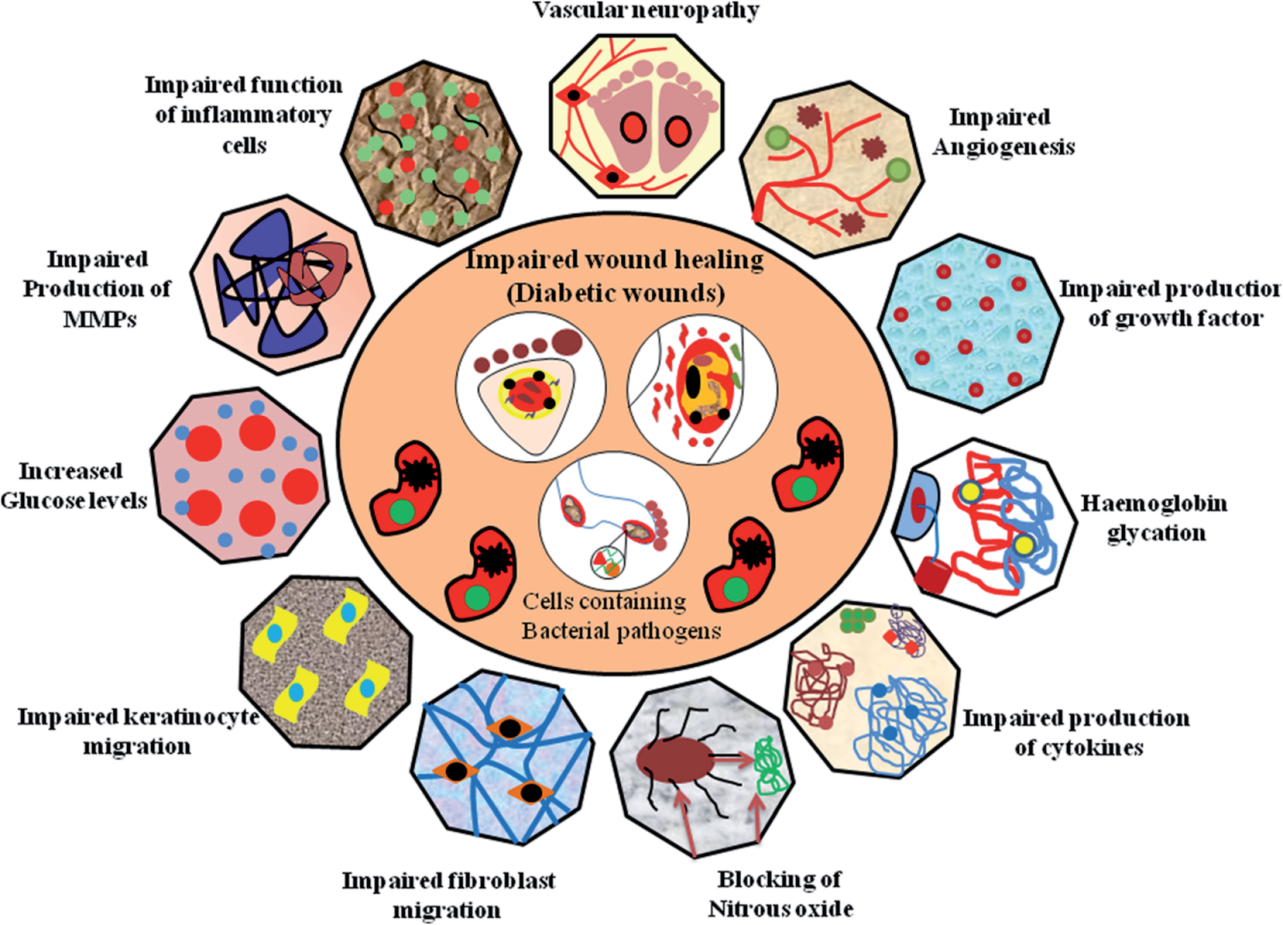
Factors responsible for diabetic wound healing process.

Diabetic foot ulcers are considered to be a multiplex mechanism involving various complications such as diabetic neuropathy (DN), peripheral vascular disease (PVD), retinopathy, myopathy and nephropathy, impairment in angiogenic response, impairment in neutrophils and macrophage function, production of pro-inflammatory cytokines, microvascular complications such as atherosclerosis, impaired production of growth factors, impaired proliferation and migration of fibroblasts and keratinocytes in diabetic wound healing models.^43,44^ In addition, blocking of nitrous oxide, impairment in inflammatory functioning of cells, hyperglycaemia, glycation of hemoglobin, impairment in production of cytokines, impairment in MMPs, impairment in accumulation of collagen, down regulation in the expression of neuropeptides together with an inflammatory response,^45^ deficiency of fibrinolysis inhibitor,^46^ PDGF modification,^47^ decreased amount of epidermal nerves and misbalance between the ECM and MMPs^48^ are few other risk factors responsible for impaired diabetic wound healing, as shown in Fig. 3.

### 3.1 Diabetic neuropathy (DN)

Patients with diabetic neuropathy (DN) are at a higher risk of developing DFUs. DN is a disease causing impairment of movement, sensations and health aspects depending on the affected nerve.^49^ Approximately 66% of individuals with diabetes suffer from peripheral neuropathy in their lower extremity. There are several factors responsible for neuropathy, namely, abnormalities in the metabolism of fatty acids,^50^ pre-diabetes neuropathy,^51,52^ protein kinase-C pathway activation,^53^ myoinisitol,^54^ formation of glycated end products,^55^ production of neural tissues,^56^ and production of growth factors.^57^ DN is also influenced by peripheral axonal degeneration, decreased blood supply, nerve conduction and segmental demyelination, culminating in callus formation.^58^ DN impairs the axon reflex of nerves and damages microcirculation in the foot, resulting in peripheral arterial disease (PAD), which deteriorates by impairing the flow of blood to the targeted site of delivery.^59^ Diabetic patients affected with neuropathy exhibit motor, sensory and autonomic divisions of the nervous system. Motor neuropathy activates atrophy in the foot muscles, causing osteomyelitis.^60^ Sensory neuropathy causes disruption in the skin integrity and provides a route for microbial invasion, resulting in unhealed wounds, which later form chronic ulcers.^61^ Autonomic neuropathy results in dysfunction of the sebaceous glands and sweat glands in the foot, leading to a predisposition to fissures. Finally, the moisturizing capability of the foot is lost to a great extent and the overlying skin becomes vulnerable to infections and breaks.^62^

### 3.2 Peripheral vascular disease (PVD)

**Fig. 4:**
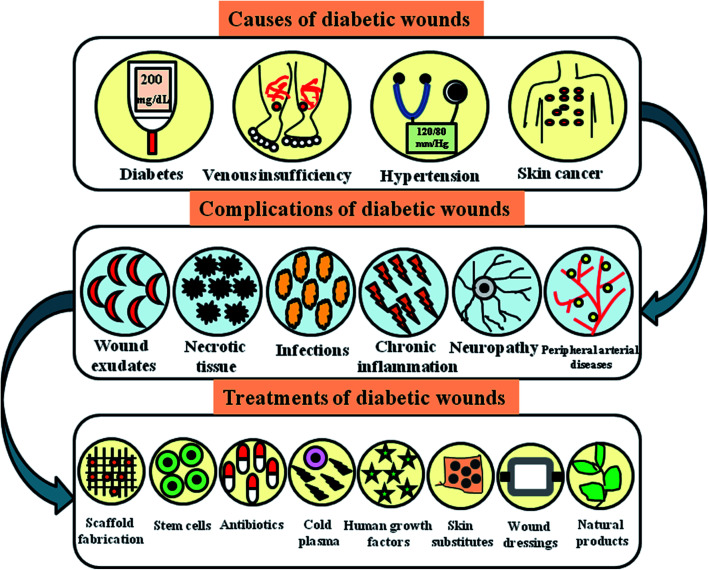
Pictorial representation showing various causes, complications and treatments of diabetic wounds.

Peripheral vascular disease (PVD) is an occlusive atherosclerotic disease of the lower extremity. Nearly 50% of patients with PVD develop foot ulcers, accounting for 70% of the death rate in type 2 diabetic patients.^63^ Diabetic patients are prone to atherosclerosis, hardening of the arteriolar walls, thickening of capillaries and endothelial proliferation.^64^ Atherosclerotic blockages of medium-sized and large arteries such as aortoiliac and femoropopliteal vessels result in chronic ischemia. In some cases, ulcers develop and progress instantly to gangrene, leading to an inadequate flow of blood.^65^ Improper blood supply to the peripheries results in impeded wound healing, which worsens the situation. The decreased amount of arterial prefusion results in the risk of infection and ulceration with impeded wound healing rates, leading to chronic problems involving amputations and gangrene.^66^ Epidemiological reports show that lipoproteins contribute to PVD. Smoking, hypertension and hyperglycemia are the significant risk factors in type 2 diabetic patients. The combination of PVD and DN leads to non-traumatic amputations.^67^Fig. 4 presents an overall view on the causes, complications and treatments of diabetic wounds.

### 3.3 Other complications

Reports indicate that a history of amputation or ulceration,^68^ peripheral edema,^69^ foot pressure,^70^ plantar callus formation,^71^ nephropathy,^72^ poor glucose control,^73^ ischemia,^74^ retinopathy^75^ and prolonged diabetes^76^ is an important predisposing factor leading to the development of DFUs. Recent studies have reported that diabetic wounds exhibit a prolonged inflammatory phase due to impairment of phagocytes and macrophages, resulting in the excessive release of MMPs (matrix metalloproteinases), causing degradation of collagen and extracellular matrix (ECM).^77^ Upon exposure to a high glucose environment, accelerated glycosylation restricts the migration and proliferation of human keratinocytes to the wound surface and contributes to impairment of the diabetic wound healing process.^78^

Current treatment approaches for DFUs include debridement, hyperbaric oxygen therapy, offloading, surgery and several wound bed formulations, which were developed to improve the rate of wound closure and wound contraction in the diabetic wound healing process.^79^ However, the development of DFUs and risk-associated amputations remains a major concern. Therefore, addressing effective management strategies is essential for the treatment of DFU.

## 4. Current therapies for diabetic foot ulcers

Progenitor cells and stem cells play a therapeutic role, which is to improve vascularization and induce angiogenesis of the ischemic limb, consequently increasing the rate of healing, relieving pain and finally protecting the limbs from amputation. Based on the origin of the tissue, stem cells can be categorized into mesenchymal stem cells (MSCs), hematopoietic stem cells (HSCs), muscle stem cells, and neural stem cells (NSCs). Stem cells prevail in several tissues and have the potential to differentiate, and hence can be exploited for DFU treatment.^80^Table 1 presents details on the different types of current DFU therapies approved by the Food and Drug Administration (FDA).

**Table 1:** Different types of DFU therapies approved by the Food and Drug Administration (FDA)

S. no	Name of therapy	Administration route	Pharmaceutical form	Merits	Demerits	Ref.
1	Cell therapy (stem cells)	Locally	Gel or injection	Stimulates various cellular mechanisms for chronic wound regeneration	Short lifetime	81
2	dermaPace system	Shock waves	Device	Stimulates wound mechanically resulting in removal of the damaged tissue	Various side effects (bruises, pain, *etc.*)	82
3	Granulox	Topical	Spray	Enhances wound healing of diabetic wounds	Short lifetime	83
4	Tazobactam/piperacillin	Locally	Injectable	Broad spectrum advantage in wound infections and results in low nephrotoxicity	Adverse side effects, which include diarrhea	84
5	Becaplermin	Topical	Gel	Stimulates growth factors in DFU treatment	Short lifetime	85
6	Collagenase	Topical	Ointment	Minimum blood loss, easy application and endothelial tissue proliferation	Exudation, burning and inflammation	86
7	Deferoxamine	Locally	Injectable	Decrease in the ulcer area with less time	Adverse side effects with low lifetime	87
8	Omnigraft	Topical	Device	Improvement in DFU treatment	Swelling, formation of new ulcers, new infections and existing ulcers may worsen	88
9	Provant	Locally	Device	Potential for pressure ulcers	Little evidence of efficacy	89

Among the DFU therapies approved by the FDA, stem cell-based therapy has emerged as an effective interventional treatment strategy used to treat DFUs, which is presently used as an alternative for amputations. Stem cells synthesize cytokines, which enhance immunomodulation, angiogenesis, cell recruitment, extracellular matrix (ECM) remodeling and neuroregeneration. Stem cells possess the capability to differentiate into various cell types including keratinocytes, endothelial cells, myofibroblasts and pericytes, which play an important role in diabetic wound healing. Ormazabal *et al.* studied the diabetic wound healing effect of secretomes derived from undifferentiated human mesenchymal stem cells, *i.e.*, human endothelial cells (hMSC-EC). The results showed that hMSC-EC promoted the proliferation of endothelial cells and *in vivo* wound healing in diabetic models. Five types of recombinant proteins including angiopoietin-2, angiopoietin-1, matrix metallopeptidase 9, fibroblast growth factor and vascular endothelial growth factor (VEGF) have been identified in hMSC-EC secretomes. These cocktail proteins enhance wound healing cascades under hyperglycemic levels.^90^ Tanaka *et al.* investigated the efficacy and safety of quantity and quality culture system (QQc)-based peripheral mononuclear cell therapy for the treatment of non-healing chronic extremity wounds. The samples were collected from 9 individuals with approximately 10 chronic wound ulcers. The wound healing cascade was observed photometrically at 2 week intervals. Six of the total 10 cases exhibited complete wound closure with an average rate of 73.2% ± 40.1% at an interval of 12 weeks. These experimental results indicated the safety and feasibility of mononuclear cell-QQc therapy in DFU patients, which is regarded as an effective vasculogenic strategy for the treatment of limb salvage.^91^

**Fig. 5:**
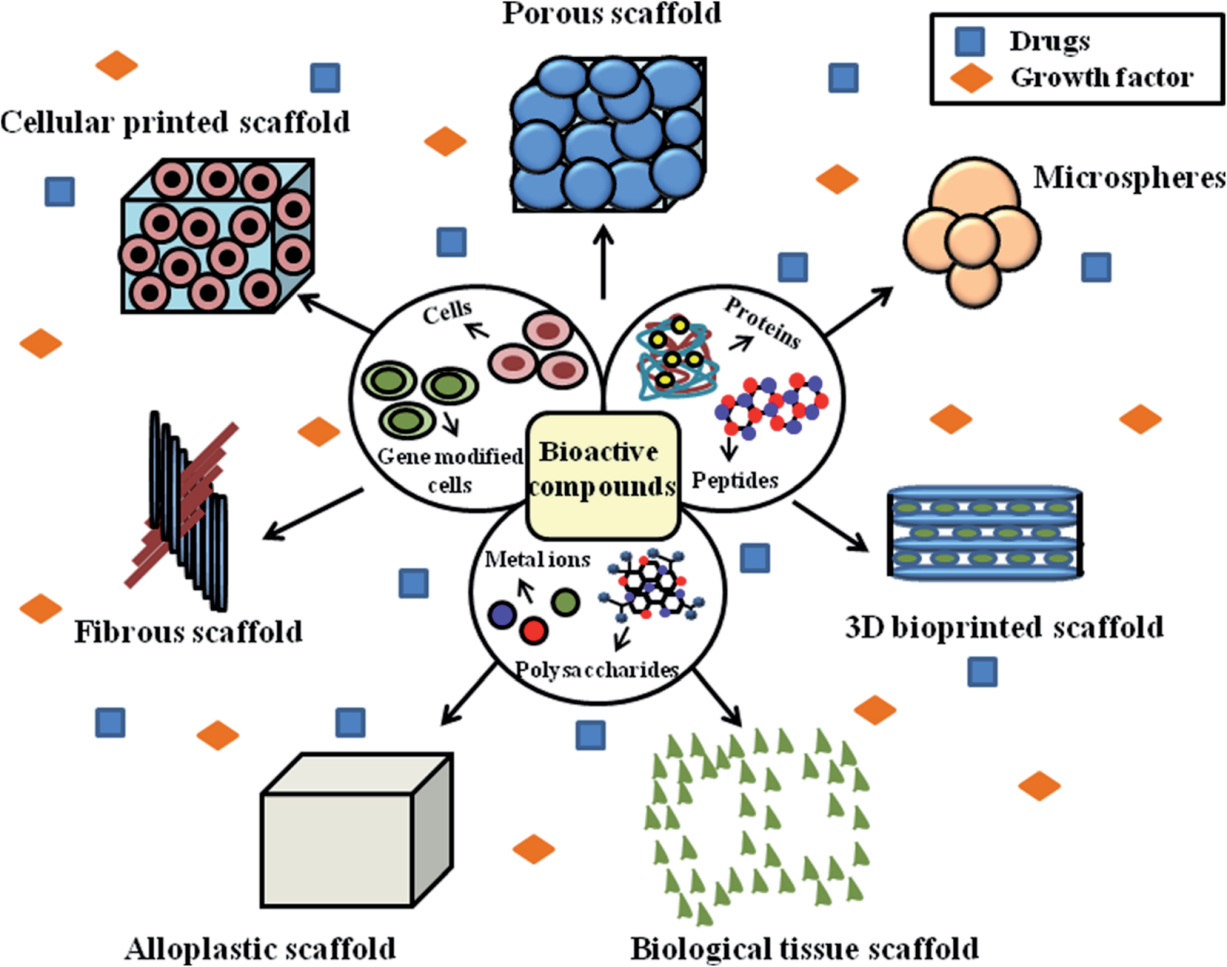
Design and manufacture of different types of scaffolds using bioactive compounds.

Growth factors serve as signaling molecules between cells. Specifically, hormones and cytokines bind to the receptors on the surface of the target cells, which enhances cell growth, differentiation and migration. Growth factor-based therapy is considered to be a highly effective method for diabetic wound healing applications. The frequently used growth factors include granulocyte colony-stimulating factor and human platelet-derived growth factor-BB. Platelet-derived growth factors are used for the treatment of neuropathic ulcers.^92^ DFU lesions are formed due to decreased levels of epidermal growth factor (EGF) and receptors. Generally, growth factors promote the proliferation of fibroblasts, peripheral nerve regeneration, neo-epidermal thickening, proliferation and differentiation of fibroblasts and gliocytes. Previous studies have reported that growth factors stimulate the synthesis of proteins by modulating signal transduction and replication of DNA and RNA of epidermal cells.^93^ Different types of scaffolds can be designed and manufactured using growth factors and bioactive compounds, as shown in Fig. 5.

Fibrous scaffolds are regarded as the predominantly used model for the treatment of chronic wounds. The reason for this may be due to the hierarchical structure of fibrous scaffolds, where their wall matrix was found to be similar to the natural ECM, their interconnected network supports tissue growth and cell proliferation, and their gross geometry was found to be similar to patient anatomical defects. Fibrous scaffolds possess a high surface area, which is about two orders of magnitude higher than that of conventional scaffolds. The higher surface area of fibrous scaffolds enhances their hydrolytic degradation and increases the amount of serum proteins, making them suitable for tissue engineering applications. Hence, the above-mentioned therapies are considered the most potent and current wound management approaches for DFU treatment.

## 5. Nanomaterial-based therapies for diabetic foot ulcers

**Fig. 6:**
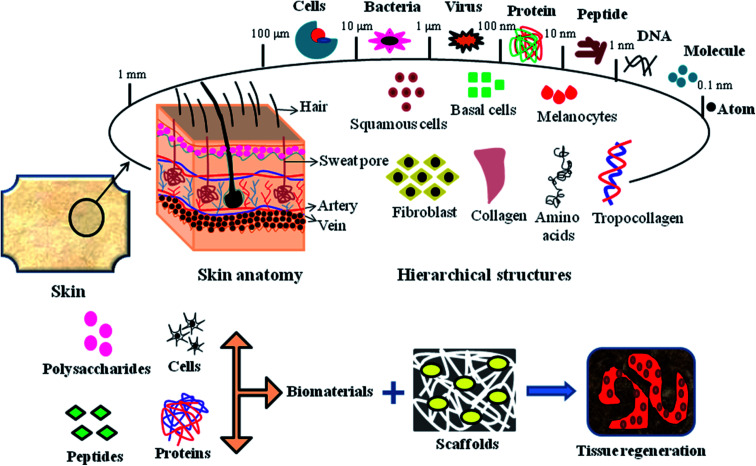
Hierarchical structure of skin tissue, emphasizing the mode of biomaterial impregnation into 3D scaffolds for tissue regeneration applications.

An ideal biocompatible wound dressing provides protection from primary and secondary infections, promotes wound tissue regeneration, removes wound exudates and provides a suitable moist environment for the skin. There are various types of commercial wound dressings for DFU treatment, which differ in material, shape, mode and method for their production. Wound dressings are regarded as medicated systems that deliver therapeutic substances such as growth factors, stem cells, drugs, peptides and bioactive substances to the targeted site. The hierarchical structure of wound tissue upon the impregnation of bioactive components, proteins, peptides and cells for treatment *via* tissue engineering is shown in Fig. 6.

Bionect, Unite Biomatrix, BGC Matrix, Promogran Prisma Matrix, Dermacol/Ag, Aquacel Hydrofiber, Regranex, Medihoney, Algisite, Sorbalgon, Kaltostat, Biatain, DuoDERM, Allevyn, Mepilex Ag and Ligasano are some of the few commercial wound dressings suitable for DFU treatment.^94^ However, although these wound dressings minimize pain and trauma to patients, maintain a moist environment, and promote granulation and vascularization to tissues, they still have some limitations such as inadequate flow of blood supply to tissues, frequent wound exudates and inefficient targeted delivery to the wound site. Nanomaterial therapeutics for diabetic wound healing can be subdivided into two main categories, as follows: (1) nanomaterials that show intrinsic characteristics beneficial for the treatment of wound healing and (2) nanomaterials that serve as drug delivery vehicles for encapsulating therapeutic agents/drugs. Nanomaterials also act as chemical angiogenic substrates to stimulate the growth of blood vessels locally.^16^ Nanomaterials stimulate angiogenic effects, allowing the formation of blood vessels in diabetic wounds, promote the migration of endothelial cells, regulate the rearrangement of the cytoskeleton, activate redox signaling and form focal adhesions.^95^ The generation of ROS has been shown in redox signaling pathways during the process of angiogenesis. The use of several nanomaterials such as nanoemulsions, nanoliposomes, nanoparticles and nanofibers have been reported in the treatment of diabetic foot ulcers to promote vascularization, angiogenesis, tissue repair, and epithelialization and in various wound tissue regeneration scenarios.

### 5.1 Nanoemulsions for DFUs

Nanotechnology-based nanoemulsion platforms show promising therapeutic delivery through topical pathways. Nanoemulsions are widely used formulations in diabetic wound healing applications owing to their physiochemical properties and high patient tolerance. The application of nanoemulsion-based therapeutics has been recently reported in diabetic wound healing approaches.^96^ Chakraborty *et al.* synthesized a topical gel formulation made up of homogenized *Aloe vera* gel incorporated with an insulin-loaded nanoemulsion using oleic acid, polyethylene glycol 400 and Tween 80 to obtain nanodroplet-sized particles. The physicochemical properties of the gel-based formulations indicated good permeation, spreadability and stability. The insulin and glucose levels in diabetic rats exhibited an antidiabetic effect (*p* < 0.001) in the case of the insulin-treated groups. The diabetic wound healing action was strongly evidenced by the increase in wound contraction (75%) with the gel-based formulations containing a combination of homogenized *Aloe vera* gel and insulin-loaded nanoemulsion. Histopathological observations revealed an improvement in the histological architecture of the tested groups. Skin irritation assays demonstrated that the gel formulation was non-irritant, non-cytotoxic and safe for topical application. Chakraborty *et al.* concluded that the synergistic effect of the insulin-loaded nanoemulsion and homogenized *Aloe vera* gel resulted in faster wound closure in diabetic rats and proved to be an effective and promising approach for the treatment of diabetic wounds.^97^ The topical application of insulin–*Aloe vera* enhanced the percentage of wound healing to a greater extent, while the nanoemulsion gel embedded with a combination of *Aloe vera* and insulin mainly contributed to the overall diabetic wound healing therapy. Thus, nanoemulsions are considered as broadly assessed topical applications in wound healing studies with respect to physicochemical properties and greater patient compliance.

Yeo *et al.* formulated a tocotrienol-rich naringenin based nanoemulgel for the treatment of diabetic wound infections. Stable nanoemulgels were assessed for droplet size, surface charge, spreadability, polydispersity index, viscosity, *in vitro* release kinetics and mucoadhesive property. They reported that an increase in the polymer concentration of the nanoemulgels increased the mucoadhesive property with a decrease in the rate of drug release. The *in vitro* release kinetic behaviour of naringenin revealed a sustained and controlled mode of release up to 74.62% ± 4.54% within a period of 24 h. Thus, the use of nanoemulgels is a promising approach in wound management associated with diabetes complications.^98^ Nanomaterial-based drug delivery paradigms using nanoemulsion platform have elucidated increased wound healing potential in therapeutic delivery *via* topical routes. These types of nanoformulations are potentially monodynamically stable and can potentiate and permeate therapeutics very easily from the rigid stratum corneum *via* the paracellular and transcellular pathways. Gundogdu *et al.* evaluated the effects of Zn-containing nanoemulsion (NE) formulations and boronophenylalanine (BFA) on diabetic wound healing rats. The MTT assay showed that 50 μM of Zn had a positive effect on cell proliferation. In the case of the scratch assay, 10 μM of BFA increased the proliferation and migration of human dermal fibroblast (HDF) cells compared to the control group. Histopathological observations proved that wound healing was complete in the case of Zn-NE and BFA compared to the untreated groups. Thus, a low concentration of BFA-containing NE gave promising evidence in diabetic wound healing with complete epithelialization and angiogenesis.^99^ Consequently, nanoemulsions with a very small droplet size, large surface area and surface tension are regarded as beneficial systems for the targeted delivery of bioactive compounds through the surface of the skin. These characteristics allow the homogeneous distribution of droplets on the skin surface and allow the easy penetration of bioactive compounds in the skin, resulting in accelerated diabetic wound healing.

Valizadeh *et al.* developed a nanoemulsion gel incorporated with levofloxacin for accelerated topical application. Scratch assays proved that the nanoemulsion gel containing levofloxacin showed a greater proliferation effect compared to the negative control. The animals treated with the nanoemulsion gel exhibited a reduction in the number of inflammatory cells and period of epithelialization with a high amount of collagen synthesis. Immunohistochemical evaluation showed the greater intensity of TGF-β and CD31 in the treatment groups on day 12 post-treatment. The skin irritation assays showed that the prepared nanoemulsion gel containing levofloxacin is suitable for topical application. Thus, Valizadeh *et al.* concluded that the nanoemulsion gel can be a promising material for diabetic wound healing by controlling the state of infection and helping to trigger the healing process.^100^ Natural oils act as the oil phase in the formulation of nanoemulsions. Drug-loaded nanoemulsions show beneficial effects on different phases of the diabetic wound healing process such as collagen synthesis, fibroplasia and wound contraction, resulting in faster wound healing potential. In conclusion, the levofloxacin-loaded sesame oil nanoemulsion can be applied as an efficient formulation for the treatment of diabetic wounds by controlling wound infections and can speed up the wound healing process. Javadi *et al.* studied the antidiabetic properties of an oil/water nanoemulsion using cumin essential oil and nettle extract in streptozotocin-induced diabetic rats. Several histological changes such as oxidative stress, apoptosis and inflammatory responses as well as the blood levels of glucose and insulin were evaluated. The essence of *Cuminum cyminum* L. and nettle nanoemulsion resulted in a decrease in the serum levels of cytokines and glucose, increased level of insulin, reduced levels of glutathione (GSH) and increased oxidized levels of superoxide dismutase (SOD), and glutathione peroxidase (GPx) in the sciatic tissue of the diabetic rats. In brief, the administration of both nettle aqueous extract and nanoemulsion after five consecutive days in diabetic rats caused a remarkable reduction in the blood levels of TNF-α, IL-1β and IL-6. Finally, the incorporation of *Cuminum cyminum* L. essence significantly decreased the blood glucose levels of TNF-α, IL-1β and IL-6 in the diabetic rats. Thus, it can be concluded that the prepared nanoemulsion acts as a potential neuroprotective agent against streptozotocin-induced diabetic rats through the modulation of inflammation, histopathological changes, oxidative stress and apoptosis. Therefore, the nanoemulsion can also be used in the treatment of diabetic neuropathy.^101^ Mahadev *et al.* evaluated and studied a quercetin nanoemulsion (Que-NE) as a drug delivery system with improved therapeutic efficacy and bioavailability in diabetic-induced rats. The droplet size of Que-NE was 125.51 nm, its polydispersity index was 0.215 and its entrapment efficiency was found to be 87.04%. Que-NE exhibited a superior mode of release and accelerated oral bioavailability compared to pure quercetin. According to the results, it can be observed that Que-NE possesses therapeutic and protective properties in managing the blood glucose level, tissue injury markers, body weight and lipid profile, and the structure of hepatocytes and pancreatic β cells are protected. Thus, the ultrasonically assisted Que-NE showed accelerated oral bioavailability and promoted protective and therapeutic antidiabetic effect.^102^

Tiwari *et al.* studied the photo-protective activity of essential oil-based microemulsions under UV-C and visible light conditions. Itraconazole drug was exposed to UV-C irradiation conditions and the photoprotection activity of clove, cinnamon, eugenol and oregano essential oils was analyzed. The antimicrobial activity against *C. albicans* showed no specific change in the ITZ-loaded microemulsion between the untreated and treated days, while the activity of the bulk drug was drastically reduced in the UV-C sample. According to the results, it can be concluded that the microemulsions act as an efficient photo-protective drug delivery system for light-sensitive compounds. Furthermore, the drug-loaded microemulsions possess favorable properties such as easy formation (spontaneous formation and zero interfacial tension), high solubilization capacity (high surface area), small droplet size, optical isotropy, and most importantly enhance the shelf-life and thermodynamic stability of the nanomaterial. These are reasons for the application of nanomaterial-based therapeutic approaches for the treatment of DFUs.^103^ Franklyne *et al.* studied the efficiency of eugenol microemulsions drug delivery vehicles loaded with triclosan, thereby preventing the selection of resistant clones. The selection of triclosan-resistant clones was determined by the broth microdilution method. Upon repeated passages with different concentrations of triclosan, mutant strains of *E. faecalis* were found to increase by 8-fold in MBC, which ranged from 250 μg mL^−1^ to 2 mg mL^−1^, and mutant strains of *S. mutans* and *S. aureus* with a nearly 8-fold increase in MBC, ranging from 125 μg mL^−1^ to 1 mg mL^−1^. These types of mutants were not expressed in the EuTT20-5-treated cultures. Therefore, EuTT20-5 not only enhanced the efficacy of triclosan, but also has equal potency against triclosan-resistant clones. They concluded that the eugenol-loaded triclosan microemulsions can be used in endodontic therapy and possess efficacy to act against resistant clones.^104^ In this particular review, we can clearly demonstrate that nanomaterials composed of drug-loaded nanoemulsions/microemulsions possess antimicrobial potential against resistant strains/clones and promote drug-targeted delivery against MDR strains. The formation of a biofilm is considered an important pathophysiological step in diabetic wounds, resulting in the development of antibiotic resistance, chronicity and progression of lesions, and ultimately delayed wound healing cascades. In this case, nanomaterials composed of nanoemulsions act as efficient tools to prevent biofilm formation in diabetic wounds, and thus result in effective re-vascularization and angiogenic potential.

### 5.2 Nanoparticles for DFUs

Nanotherapeutic-based approaches including nanoscaffolds and nanoparticles with a size in the range of 1–100 nm are promising strategies for enhanced diabetic wound healing applications. Nanoparticles possess a small size and great surface area to volume ratio, which significantly increase the penetration and biological interaction at the wound site. Nanoparticles are ideal for topical drug delivery applications, eliciting cell proliferation, cell signaling, cell-to-cell interactions, vascularization and epithelialization for enhanced wound healing approaches.^105^ Silver nanoparticles (AgNPs) have been extensively used in wound therapy, especially chronic wounds (diabetic wounds). Table 2 presents details on the various nanoparticle-mediated therapeutic approaches for diabetic wound healing.

**Table 2:** Nanoparticle-mediated therapeutic approach for diabetic wound healing

S. no	Types of nanoparticles	Route of administration	*In vitro* model	*In vivo* model	Inferences	Ref.
1	NLC and SLN nanoparticles	Topical NLC-rhEGF and SLN-rhEGF dressing models at the wound site	Keratinocytes, fibroblasts	Thickness of 8 mm diameter wound was created in male diabetic db/db mice	rhEGF-associated lipid nanoparticles reveal higher proliferation of keratinocytes and fibroblasts and greater reepithelialization compared to normal rhEGF	106
2	NaCMCh nanoparticles	Topical delivery of nanoparticles loaded with chitosan hydrogel at site of delivery	Fibroblast	Thickness of 20 mm diameter wound was created in Sprague-Dawley diabetic rats	Cells treated with nanoparticles showed greater cell viability with increased rate of wound healing compared to normal rhEGF	107
3	AuNPs	Topical delivery of gelatin hydrogel impregnated with KGF-AuNPs	Keratinocytes	Thickness of 10 mm diameter wound was created in diabetic rats	KGF-AuNPs resulted in enhanced healing effect compared to normal KGF and promoted wound closure and reepithelialization together with the expression of α-SMA, Col-I and TGF-β1, resulting in accelerated wound healing in comparison to the controls	108
4	Gelatin nanoparticles	Topical delivery of drug associated hyaluronic acid/collagen nanofibrous mats at site of delivery	Human endothelial cells	Thickness of 15 mm diameter wound was created in Sprague-Dawley male diabetic rats	Gelatin nanoparticles associated with growth factors showed enhanced wound healing rate, cell proliferation, vascularization and reepithelialization compared to controls	109
5	AgNPs loaded with ε-polylysine nanocomposites	Topical delivery of nanoparticles with antibiotic load to the wound site	Fibroblast cells with 80% cell viability post-treatment of 2 days with the nanoparticles	Thickness of 1.5 cm diameter wound created in Wistar albino rats followed by inoculation of *S. aureus* and *P . aeruginosa*	Nano-biocomposite resulted in the acceleration of wound healing without adverse side effects on the tissues of the dermal layer, eliminating wound infections	110
6	AuNPs	Topical delivery of nanoparticles consisting of antioxidants on the wound site	Nanoparticles loaded with antioxidants significantly decreased the expression of RAGE in fibroblast cells	Thickness of 1 cm diameter wound created in the BALB/c diabetic mice	Nanoparticles encapsulated with antioxidants increased the percentage of diabetic wound healing by decreasing the expression of RAGE compared to the free antioxidant and control group	111
7	FNPs	Topical delivery of wound bandages with antibiotic loaded to the wound site	Toxicity of bandages determined against human fibroblast cell lines, which proves its cyto-compatibility	Thickness of 1.5 cm diameter excisional wound created in Sprague-Dawley diabetic rats by inoculation of *E. coli*, *S. aureus* and *C. albicans*	Bandages made up of nanoparticles showed reduction in the microbial area, resulting in accelerated rate of wound healing	112
8	AUNC-L	Topical delivery of nanoclusters to the wound site	Cyto-compatibility of nanoclusters with human fibroblast cells, which showed greater cell viability compared to ampicillin	Thickness of 1.5 cm diameter wound created in male diabetic Wistar albino rats followed infection with MRSA	Synthesized nanoclusters eradicated infections, exhibiting rapid wound healing potential	113
9	Cationic lipid nanoparticles	Topical delivery to the wound site	Knockdown of LPP-10 associated with protein expression, an important factor in cell integrity	Thickness of 10 mm diameter wound created in diabetic rats	Nanoparticles restored antioxidant function, which helped to enhance tissue regeneration and augment homeostasis in the wound environment	114

Among the various polymers available for the fabrication of polymeric nanoparticles, PVA is the most extensively used synthetic polymer, which possesses desirable properties, as follows: (1) well-described methods and formulations for its production adapted for drug delivery, ranging from micro to macromolecules and (2) protection of drugs from degradation and mode of controlled and sustained release.^115^ Azlan *et al.* biosynthesized gold nanoparticles (AuNPs) using hot and cold water extracts of *Lignosus rhinocerotis* loaded with PF127 gel for diabetic wound healing applications. The groups treated with the PF127 gel and AuNPs showed faster wound closure compared to the positive control. The treated groups showed accelerated blood vessel density and decreased number of inflammatory cells. Compared to the positive control, the vascular endothelial growth factor (VEGF) and higher prostaglandin E2 and VEGF-A levels indicated the effectiveness of DsiRNA by enhancing vascularization and production. Gram-positive bacteria such as *Corynebacterium* and *Staphylococcus* and Gram-negative bacteria such as *Rodentibacter*, *Acinetobacter* and *Pseudomonas* were found to be sensitive to the PF127 gel. They concluded that the AuNPs loaded with PF127 gel are a promising material for dressing diabetic wounds given that they promote complete vascularization and epithelialization.^116^ AuNPs were biosynthesized using hot and cold water extracts of *Lignosus rhinocerotis*, which resulted in an increase in the density of blood vessels and decrease in the amount of inflammatory cells. Here, the extract of *Lignosus rhinocerotis* acted as a therapeutic agent embedded in a nanomaterial, which can be used for diabetic wound healing applications. Although this organic extract acts as a reducing and stabilizing agent for the biosynthesis of nanoparticles, the yield efficiency of the nanoparticles is higher and they can exhibit a less cytotoxic response to living cells.

Chen *et al.* prepared an injectable multifunctional composite hydrogel using cerium-based bioactive glass (Ce-BG) transformed into a gelatin-based methacryloyl (GelMA) hydrogel. The prepared Ce-BG/GelMA hydrogel promoted the migration of endothelial cells and exhibited excellent cytocompatibility. The *in vitro* antibacterial assays showed that the 5 mol% CeO_2_-based bioactive glass/GelMA composite hydrogel exhibited good antibacterial properties. The *in vivo* studies proved that the CeO_2_-based composite hydrogel improved the healing properties in diabetic rats by enhancing the deposition of collagen, angiogenesis and formation of granulation tissue. Thus, the production of multifunctional hydrogels with angiogenic and antibacterial properties is a promising strategy to promote diabetic wound healing applications.^117^ Generally, hydrogels provide temporal and spatial control over the release of therapeutic agents, which include small molecules/drugs, cells and macromolecular drugs. Based on their tunable properties and rate of degradability, hydrogels act as efficient platforms to carry therapeutic drugs and control the mode of their release. Hydrogels as nanomaterials enriched with anti-inflammatory and angiogenic properties, resulting in a controlled and sustained mode of release, possess therapeutic potential for the treatment of diabetic wounds. Suresh *et al.* synthesized AgNPs using *Turbinaria conoides* aqueous extract (TCAgNPs). Characterization confirmed the presence of AgNPs with an absorption at 452 nm and the obtained particles were spherical and polydisperse in nature. The TCAgNPs showed excellent antibacterial activity against multidrug-resistant strains in DFUs such as *Klebsiella pneumoniae*, *Enterococcus faecalis*, *Staphylococcus aureus* and *Pseudomonas aeruginosa* based on the minimum inhibitory concentration and disc diffusion method. They concluded that TCAgNPs can be regarded as an efficient healing strategy for diabetic wound infections.^118^ The aqueous extract of *Turbinaria conoides* acts as a reducing and stabilizing agent in the synthesis of silver nanoparticles. This particular extract possesses therapeutic potential and acts as an effective nanomaterial against Gram-positive and Gram-negative strains (both susceptible and resistant strains). Furthermore, although the reducing agent is an organic compound, the level of toxicity would be much less compared to chemically synthesized nanoparticles. Essa *et al.* evaluated the effectiveness of AgNPs (SlivrSTAT) by enhancing the wound healing rate in non-ischemic DFU patients. The wound healing rate of the SlivrSTAT group was higher than that of commercially available wound dressing models. Therefore, the SlivrSTAT Gel-based wound dressing is considered as an efficient model for DFU treatment.^119^ AgNPs possess broad-spectrum antimicrobial efficacy because of their intrinsic therapeutic characteristics and multisite action. The antibacterial properties of AgNPs make them effective materials for use in wound dressings, anti-neoplastic drug delivery and artificial implantation. AgNP nanomaterial act by destroying the bacterial cell wall membrane and causing cellular disintegration of resistant strains in diabetic wounds. Thus, AgNPs show anti-inflammatory and antibacterial effects, and therefore improve the rate of healing of ulcers and wounds.

The particle surface area, shape and size are the material characteristics considered from a toxicological point of view, given that the interaction between biological organisms and nanomaterials specifically takes place at the surface of nanoparticles. The surface area of NPs exponentially increases as their particle size decreases and a greater proportion of particles, molecules or atoms will be greatly exposed on the surface rather than the bulk of nanomaterials.^120^ The lung is considered as effective barrier against the distribution and uptake of NPs. In the route of the human respiratory tract, inhaled particles of different sizes reveal fractional depositions, given that ultrafine nanoparticles smaller than 100 nm are deposited in all the regions, while particles less than 10 nm are deposited specifically in the tracheobronchial region, and particles between 10 and 20 nm deposit specifically in the alveolar region. The toxicity of NPs arises from their size-related ability to enter the biological system and modify the protein structure through the formation of NP and protein complexes, resulting in the degradation of proteins.^121^ Nanoparticle size has an important effect on the route and rate of clearance from the body, especially present in parenteral dosage forms. NPs less than 50 nm can be easily administered by means of intravenous injection, which are found to be potentially toxic and disperse easily and quickly through the cells/tissues, accumulating in the heart, liver, kidney, blood, lungs, spleen, thymus and reproductive organs. Larger NPs greater than 100 nm are present in reticuloendothelial system tissues but not as much as smaller particles. Thus, nanoparticle size plays an important role in *in vitro* and *in vivo* cytotoxicity systems and it can be concluded that the smaller the size of nanoparticles, the greater their toxicity to living cells. The size effects of different nanomaterials with respect to toxicological responses are shown in Table 3.

**Table 3:** The impact and effect of nanoparticle size on toxicity to living systems

Nanomaterial	Species	Study parameters	Toxicity study	Toxicity mechanism	Administration route	Ref
Silica	Mouse	Distribution in tissues and excretion in feces and urine	Immunohistochemistry	Particle size ranging from 100 and 200 nm shows inflammatory response in the liver. All NPs remained aggregates through macrophage trapping in the spleen	Intravenous	122
Gold	Mouse, zebrafish	Distribution of size-dependent particles in the lung, kidneys, liver, spleen and brain	Percent mortality	AuNPs with a size ranging from 3, 10, 50 and 100 nm possess less toxicity	Exposure of zebrafish embryo to NPs and intravenous (mouse)	123
Silver	Mouse	Distribution in the body	Histopathology measurement of cytokines, immune phenotyping and blood biochemistry	Inflammatory responses, organ toxicity, distribution of increased B cells, and production of cytokines and inflammatory cell infiltrates	Oral	124
Polystyrene	Rat	Protein assay	LDH assay	Reactive oxygen species	Instillation and sub-conjunctival	125
Copper	Mouse	Changes in plasma electrolyte content and blood gas of the copper elements in serum, renal tissues and urine	Biochemistry analysis (serum copper, ceruloplasmin and urine copper)	Accumulation of alkalescent substance	Oral	126
TiO_2_	Mouse	Up-regulation of placenta growth factor and chemokines (CXCL5, CXCL1 and CCL3)	Micro-array gene expression, morphometric and pathway analyses	Macrophages accumulation, type II pneumocyte hyperplasia, pulmonary emphysema, extensive disruption of septa and apoptosis of epithelial cells	Intratracheal	127
PLGA	Mouse	Histopathology assays	Tissue distribution, histopathology assays	No toxicity	Oral	128

The aspect ratios and shapes of particles are considered as the key factors that influence the toxicity of NPs. Nanomaterials have various shapes including spheres, rings, fibers, tubes and planes. The *in vivo* toxicity of nanomaterials has an adverse effect on the endocytosis or mode of clearance by macrophages, given that their shape can determine the membrane wrapping process during phagocytosis and endocytosis. Cylindrical, disc-like and hemispherical particles outperform spherical particles by evading uptake by phagocytic cells, and consequently non-spherical particles flow through the capillaries and get adhered to the blood vessels, causing many other biological consequences. If NPs are found to be biologically persistent, chronic inflammation leads to mutagenic events, resulting in the formation of mesothelioma. After administration *via* the intra-tracheal route, SWCNTs induced lung granulomas, and multifocal granulomatosis lesions without cell proliferation, inflammation or cytotoxicity induced a potential mechanism of pulmonary injury and toxicity.^129^ The accumulation of macrophages attempts to phagocytose retained fibers, resulting in phagocytosis. The macrophages release oxidants and cytokines, resulting in further fibrosis, inflammation and genotoxicity to the mesothelial cells in the regions of congestion around the stromal entrances. Nickel NP clusters consisted of 60 nm particles, which exhibited higher toxicity in zebrafish compared to spherical NPs, indicating that the differences in aggregation and shape are responsible for accelerated toxicity. Thus, the nickel NPs found in cluster form adhered readily and were retained for an extended period in the intestinal lumen, increasing the cellular stress.^130^ In the case of TiO_2_ NPs, it was determined that fibrous structures with greater ratios were more cytotoxic than spherical structures. Specifically, TiO_2_ NPs with a length of 15 mm were found to be extremely toxic compared to fibers with a length of 5 mm and further initiated an inflammatory response due to the presence of alveolar macrophages in mice.^131^ In conclusion, the role of the shape effects in nanotoxicity response is shown in Table 4.

**Table 4:** The impact and effect of nanoparticle shape on toxicity to living systems

Nanoparticle shape	Nanoparticle	Toxicity mechanism	Physiological responses	Ref
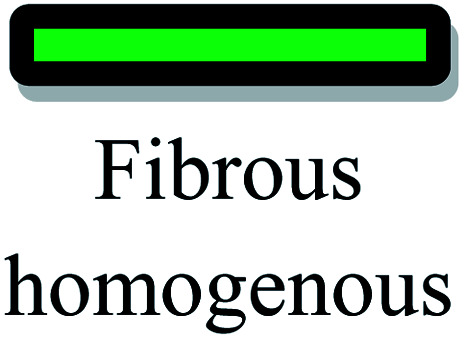	Gold, TiO_2_, SWCNT, and mesoporous silica	Membrane disruption and internalization. Severe influence on phagocytosis. Highest distorting force among the shapes. Blockage of transport channels. Smaller ratio results in faster internalization	Chronic inflammation due to mutagenic events, frustrated phagocytosis and mesothelioma formation	129 and 131
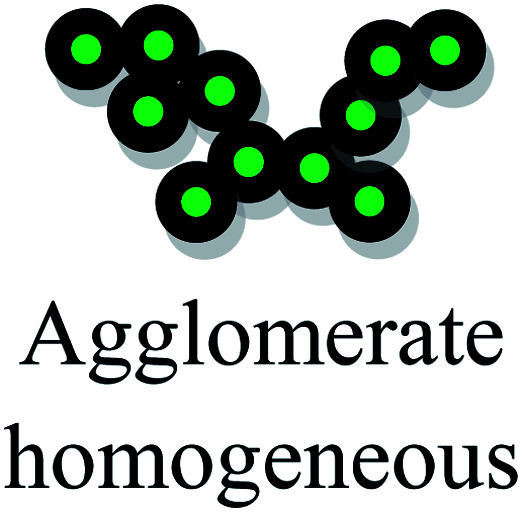	Carbon black, nickel and TiO_2_	Agglomeration or aggregation changes particles, thus increasing their visibility to macrophages	Retention time of NPs, aggregation changes and changes in size may decrease or increase toxicity	130 and 131
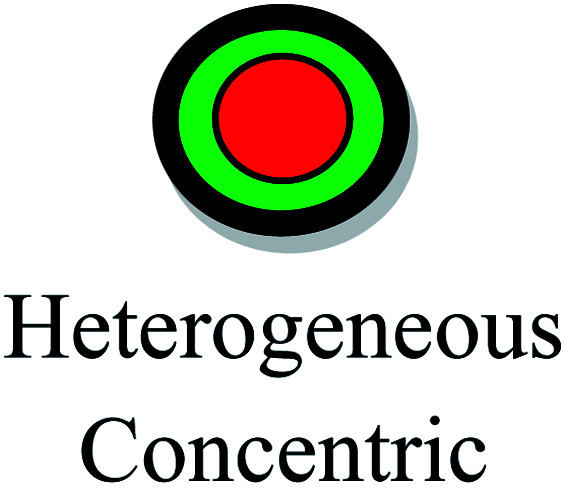	Quantum dots	Similar to spherical NPs	Similar to spherical NPs	132
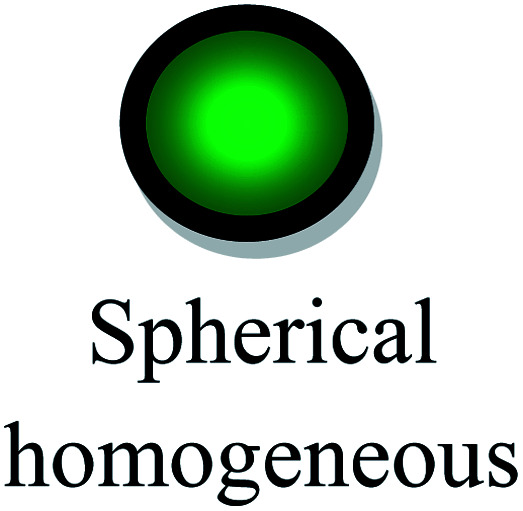	Gold, iron oxide	Membrane disruption and internalization. Higher uptake of cells with less disruption among the shapes, and least shape-dependent toxicity	Dysfunction of cell division and cellular trafficking, and mechanical interference with DNA and mitotic spindle	133
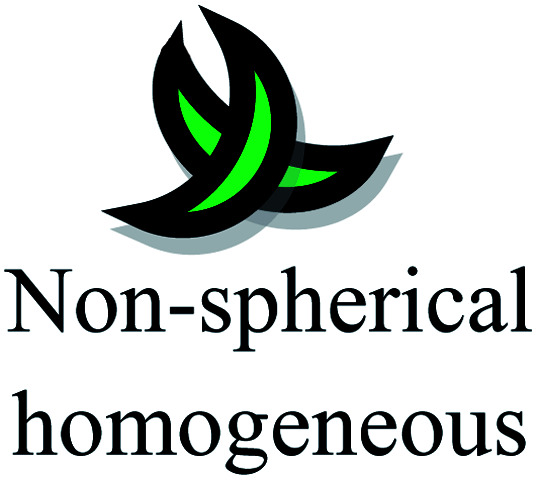	Gold	Dependent on radius of curvature. Membrane integrity disruption and transport may occur	Toxicity due to impaired phagocytosis and chronic inflammation	134
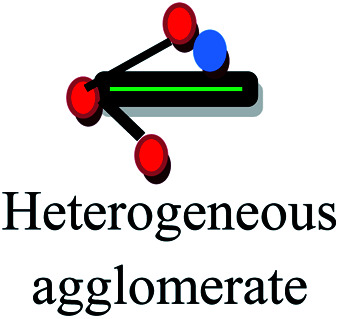	Iron oxide and ZnO	Disruption of cell membrane and aggregation dependent on prevalence of high aspect ratios	Combinatorial effect similar to the fibrous particles and aggregated particles	135

**Fig. 7:**
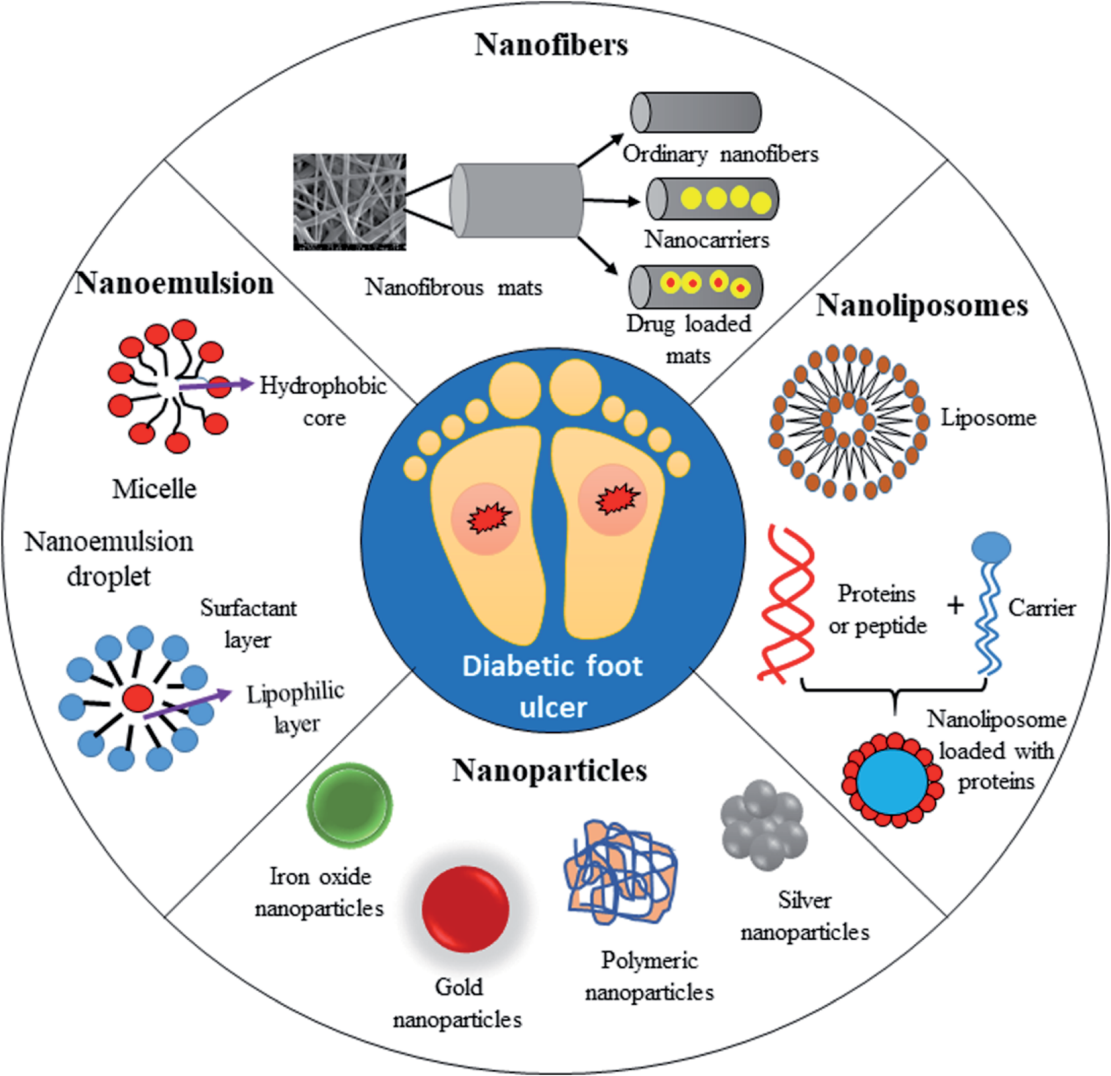
Application of different types of nanomaterials for the treatment of diabetic foot ulcers (DFUs).

Fig. 7 demonstrates the application of different nanomaterials for the treatment of DFUs. Nanoparticles can be synthesized *via* organic and inorganic methods. Organic modes (biological synthesis) for the synthesis of nanoparticles result in potent, rapid and broad-spectrum antibacterial activity against Gram-negative and Gram-positive strains. Efficient nanomaterials can be formulated using different types of biologically synthesized nanoparticles and show a sustained and controlled mode of release of Ag^+^ ions in the simulated wound environment. Biologically synthesized nanoparticles show much less toxicity to living cells, and thus act as efficient nanocarrier systems for targeted drug delivery.

### 5.3 Nanoliposomes for DFUs

Nanoliposomes are regarded as robust nanocarriers for biomedical applications because of their patient compliance, safety and quick action. Nanoliposomes are innovative technology for the purpose of encapsulation and drug delivery.^136^ Nanoliposomes are biodegradable and biocompatible in nature and have been extensively used in a wide variety of nanotherapies such as cancer therapy, cosmetics, agriculture, diagnosis, gene delivery and food technology. Kotwal *et al.* explored the molecular mechanism of nanoliposomes by comparing ATP nanoliposomes and control nanoliposomes. The isolation of total RNA using RNA seq technology revealed the overexpression of noncoding RNAs. The U1 snRNA and U1 spliceosomal RNA were upregulated with ATP nanoliposomes post treatment. Therefore, the spliceosomal RNA helped to speed up the splicing process using transcription and facilitated the transformation of pre-mRNA to mRNA. Increased functional RNA can be transformed to increase the amount of proteins, enhancing reepithelialization, neovascularization, proliferation and macrophage polarization. Thus, the accelerated ATP level triggers molecular events, resulting in increased wound healing efficacy.^137^ The experimental results clearly showed that ATP plays an important role in the wound healing process and functions as an energy-delivering molecule. Thus, ATP helps in targeting living cells and helps in drug delivery systems. The present report showed that the synthesized microRNAs regulate the cellular ATP levels to target the mitochondrial energy metabolism. The significant overexpression of noncoding RNAs resulted in accelerated diabetic wound healing process. Thus, nanoliposome-based therapeutics show a potential involvement in the extracellular matrix, which makes the wound site more accessible towards macrophages and found to be more amenable to the proliferation of macrophages. The nanoliposome-encapsulated ATP overcomes the natural barrier to the cellular entry of ATP imposed by the cell membrane.

Antimicrobial peptides (AMPs) are considered small molecules and host defense peptides for treating microbial infections. The activity of AMPs against Gram-negative and Gram-positive strains has been exploited to kill multidrug-resistant (MDR) strains and bacteria.^138^ Umar *et al.* developed a water-soluble chitosan spray containing hEGF-based liposomes, which acted as a potential wound dressing model. The viscosity, pH and particle size of the hEGF-based liposomes were found to be stable for a month. The wound healing efficacy of the hEGF-based liposomes revealed that the percentage of wound closure was significantly higher on day 6 compared to the control group. Therefore, Umar *et al.* concluded that water-soluble chitosan-containing hEGF-based liposomes can be considered a potential wound dressing for diabetic wound healing applications.^139^ Human epidermal growth factor (hEGF) possesses excellent wound healing efficacy. The incorporation of liposomes as carriers and coatings protects hEGF from enzyme degradation, immune reactions and chemical reactions. The liposome coated with hEGF showed localized and increased drug delivery to the wound site. Liposomes increase the therapeutic index and efficacy of the drug. Liposomes are generally non-toxic and non-immunogenic for non-systemic and systemic administrations to the wound site, and thus help in targeted drug delivery due to their biocompatibility and biodegradability. Eid *et al.* tailored citicoline-chitosan-coated liposomes (CT-CS-LPs) for efficient wound healing in diabetic rat models. The formulated CT-CS-LPs showed a mean size of nearly 211.6 nm with an entrapment efficiency of 50.7% and surface charge of nearly 32.1 mV. The optimized nanomaterials possessed a sustained and controlled mode of release in the simulated body fluids. The *in vivo* studies show that the optimized CT-CS-LPs enhanced the wound healing process in diabetic rats by accelerating re-epithelialization, fibroblast proliferation, reducing inflammation, angiogenesis, and connective tissue remodeling, thus leading to rapid and quick wound closure and wound contraction. Thus, the chitosan-coated nanoliposomes containing citicoline have emerged as a potential approach for enhancing the diabetic wound healing process. At all specific stages of the wound healing process, the treatment with CT-CS-LPs revealed higher VEGF immunoreactions compared to the control group and helped in the induction of fibroblast formation and micro-vessels and enhanced the epithelialization process.^140^ Generally, nanocarriers can protect drugs from degradation, increase intracellular absorption, allow the prolonged release of medication, and optimize the position of the drug at the wound site through targeting properties. These liposomes are an excellent option for transporting hydrophilic molecules such as charged and small compounds to the targeted site, acting as efficient nanocarrier therapeutics for wound healing applications. Nanoliposomes can be encapsulated with daptomycin, which helped to inhibit *S. aureus* biofilm growth compared to the intravenous administration of daptomycin for treating subcutaneous infections in a rat model.^141^ Liposome-based nanoformulations are regarded as excellent carriers for antibacterial drugs given they can mimic the structure of the bacterial cell wall membrane, prolong the drug circulation time and accelerate the uptake of cells, making them competent drug delivery systems for wound healing applications. These nanoliposomes are generally vesicular structures containing an internal aqueous compartment surrounded by a phospholipid bilayer. Nanoliposomes are considered safe, non-toxic, biodegradable, biocompatible and possess efficiency to encapsulate both lipophilic and water-soluble substances, which can serve as excellent nanomaterials for enhancing the wound re-epithelialization, vascularization and angiogenic potential in chronic wounds.

### 5.4 Nanofibers for DFUs

The electrospinning technique has been extensively used in the biomedical field to prepare biopolymeric nanofibers incorporated with organic molecules or drugs for the treatment of burns, cuts, wound healing and chronic (diabetic) ulcers. Electrospun nanofibers (ESNs) have seen the development of new generations of novel nanofibers such as composite, blend, hybrid and core–shell nanofibers, possessing mechanochemical and physicochemical characteristics that provide distinct advantages for the treatment of diabetic wounds.^58^ Nanofiber-based scaffold wound dressings have immense applicability and popularity in the biomedical field. Nanofibers ensure gaseous and nutrient exchange between damaged tissue and the environment, facilitating the absorption of exudates from the wound site.^142^Table 5 presents details on the different types of electrospun nanofibers (ESNs) incorporated with therapeutics as wound dressing models for DFUs.

**Table 5:** Electrospun nanofibers (ESNs) incorporated with therapeutics as wound dressing models

S. no	Nanofiber components	Impregnated therapeutics	Functions	Wound types	Ref
1	Manuka honey (MH)/cellulose acetate (CA)	—	Antimicrobial activity	Burn wound infections	143
2	Poly (*l*-lactide) (PLLA)/chitosan	Graphene oxide	Antibacterial activity	Chronic wound infections	144
3	Polyester urethane/CA	Polyhexamethylenebiguanide (PHMB)	Antimicrobial activity	Cut wounds	145
4	Polyvinyl alcohol (PVA)/chitosan	Nanobioglass (nBG)	Antimicrobial activity, biocompatibility	Tissue regeneration effect for infected wounds	146
5	Polyhydroxyalkanoate (PHA)	Graphene–silver nanoparticles (GAg)	Antibacterial activity	Chronic wounds	147
6	Poly(3-hydroxybutyrate-co-3-hydroxyvalerate) (PHBV)	Cerium oxide nanoparticles	Angiogenic and antioxidant properties	Chronic wounds	148
7	Cellulose/PHBV	ZnO nanocrystals	Antimicrobial activity	Infected and acute wounds	149
8	Polylactides (PLA)	AgNPs	Antibacterial activity	Chronic ulcers and burn wounds	150
9	PLA	Doxycycline (DCH)	Antimicrobial activity	Diabetic ulcers and chronic wounds	151
10	PLLA	Curcumin	Antioxidant and anti-inflammatory effects	Burn wounds	152
11	PVA/PLA	Connective-tissue growth factor (CTGF)	Anti-inflammatory and angiogenic properties	Diabetic wounds	153
12	Polydopamine/PLGA	Ponericin G1, fibroblast growth factor (FGF)	Cell proliferation and antibacterial activity, which helps to promote tissue regeneration	Burn wounds	154
13	Hyperbranched polyglycerol (HPG)/PLA	Curcumin	Anti-inflammatory, antioxidant and anti-infective properties	Burn and cut wounds	155
14	PHBV	Curcumin	Anti-tumor property, antibacterial and antioxidant and anti-inflammatory properties	Pressure ulcers, burn wounds, venous leg ulcers and diabetic wounds	156
15	Keratin/chitosan/polycaprolactone (PCL)	*Aloe vera* extract	Antibacterial, anti-inflammatory and anti-viral properties	Burn wounds and acute wounds	157
16	Gelatin/PLGA	Gentamicin sulfate and human epidermal growth factor (HEGF)	Angiogenic property and antibacterial activity	Diabetic wounds	158

Meamar *et al.* studied the delivery of doxycycline (DOX) as an inhibitor drug and venlafaxine (VEN) in bacterial cellulose (BC) nanofibers for alleviating neuropathy and inflammation in DFU patients. The formulated nanomaterial showed a loading efficiency of 48% ± 1.9% for VEN and 37.8% ± 1.6% for DOX. The size of diabetic ulcers showed a quicker reduction after a period of 12 weeks in the test group compared to the control group. Microscopic analysis showed a large amount of chronic polymorphonuclear inflammatory cells and the formation of a capillary bed at the wound site. Therefore, BC nanofibers loaded with VEN and DOX may reduce neuropathy and speed up the healing process in diabetic patients.^159^ Most wound dressings create a suitable microenvironment for the wound healing process, and thus facilitate a moist environment that removes exudates, enhance antibacterial effects, and stimulation, proliferation and migration of keratinocytes and fibroblasts at the injury site. The core part of nanofibers contains inhibitor drugs (doxycycline and venlafaxine) surrounded by a sheath part consisting of synthetic and natural polymers. Bacterial cellulose nanofibers are regarded as the purest form of cellulose. They possess unique characteristics such as high capacity, high mechanical properties, biocompatibility, 3D fibrillary network and biodegradability. Bacterial cellulose-based nanofibers loaded with drug inhibitors play a pivotal role in transdermal skin delivery, prevent wound infections, and thus enhance the wound healing process. Agarwal *et al.* prepared curcumin and silk fibroin-based nanofibers impregnated with polyvinyl alcohol (PVA) and polycaprolactone (PCL), which helped to enhance the diabetic wound healing characteristics of the nanofibers. The microscopic results showed the uniform distribution of nanofibers with a diameter in the range of 200 and 350 nm and tensile strength in the range of 12.41 to 16.80 MP. The *in vivo* experiments using nanofibers in streptozotocin-induced diabetic models exhibited a greater wound healing efficacy compared to conventional formulations. Thus, curcumin and silk fibroin-based nanofibers appear to be a promising nanomaterial candidate that exhibits anti-inflammatory and antioxidant properties for the treatment of diabetic wounds.^160^ Curcumin is a naturally occurring compound that possess wide range of therapeutic activities including anti-oxidant potential, diabetic wound healing efficacy, anti-inflammatory and anti-cancer activity. However, due to the poor solubility of curcumin, silk fibroin, which is a naturally occurring polymer, was blended with curcumin and fabricated into beadless and smooth nanofibrous mats. Polycaprolactone/polyvinyl alcohol was used as a synthetic polymer for accelerating the mechanical strength of the nanofibers. The *in vitro* release of the nanofibers possesses a sustained and controlled mode of release with prolonged duration. Thus, due to the improved bioavailability and pharmacological activity of the synthesized curcumin-loaded silk fibroin nanofibers, they act as a unique drug delivery platform for diabetic wound healing applications.

Anand *et al.* fabricated a multifunctional biomimetic scaffold (polyvinyl alcohol (PVA) – silk fibroin (SF) – sodium alginate (SA)) incorporated with asiaticoside for the diabetic wound healing process. The SEM results showed that the diameter of the fibers was in the range of 100–200 nm and their tensile strength was in the range of 12.41–16.80 MPa. The crosslinked nanofibers produced a sustained and prolonged mode of release in the biological system. The water absorption capability of the asiaticoside-loaded nanofibers increased due to the hydrophilic nature of PVA, which allowed water to diffuse in the nanofibers. After the crosslinking process, the hydrophobic nature of the nanofibers hindered water molecule transport in the nanofiber mat after a duration of 3 h, which resulted in a decrease in the water uptake capacity of the nanofibrous mats. The crosslinked nanofibers allowed prolonged release of the active drug compound from the tight cores of the fiber, maintaining the therapeutic response for a longer period. The antibacterial activity of the asiaticoside-loaded nanofibers showed a clear zone of inhibition against the *S. aureus* and *P. aeruginosa* bacterial strains. The results suggested that the antibacterial activity of the nanofibers can be enhanced by increasing the drug concentration in the nanomaterial formulations. The wound scratch assay and MTT assay on HaCaT cells confirmed significant cell migration and cell proliferation as well as low cytotoxicity levels. The number of cells constantly increased throughout the 24 h of growth, indicating that the nanofibers were non-toxic, depending on the optical density of the MTT assay. The wound healing rate of the nanofiber formulations was quantified on days 0, 3, 6, 9 and 14 days, which was found to be higher compared to the control groups. The larger surface area to volume ratio resulted in the increased absorption of exudates, associated with effective diabetic wound healing applications. The porous structure of the nanofibers were found to be biomimetic to the extracellular matrix of the skin surface, allowing cell respiration and maintaining the oxygen and water permeability at the wound site, which are essential for effective wound healing applications.^161^ The therapeutic drug asiaticoside possesses anti-oxidant, anti-inflammatory and angiogenic potential for diabetic wound healing applications. Thus, this drug was incorporated with bioactive polymers to fabricate multifunctional biomimetic nanofibrous membranes, which served as nanomaterial-based therapeutics for diabetic wound healing applications. Nanofiber-based wound dressings show greater potential to achieve complete and rapid healing of diabetic wounds, affording unique characteristics such as nanoscale structure, removal of wound exudates, large surface area, provide ECM, maintain porosity and enhance tissue regeneration properties. These properties play an essential role in cell attachment, migration and proliferation, resulting in a significant improvement in diabetic wound healing models.

Sethuram *et al.* fabricated eugenol microemulsion loaded with silver nanocomposite electrospun nanofibrous mats as wound dressings. Homogeneous and well-oriented electrospun nanofibrous mats with a pore diameter of 404.1 nm and elemental composition of 13.93% were observed. The eugenol microemulsion loaded with silver nanoparticles exhibited the highest antimicrobial efficacy against *S. aureus*. The nanofibers exhibited a sustained and controlled mode of release of silver ions in the simulated wound system. The percentage cell viability and percentage red blood cell breakdown in the human biological system were found to be 69.81% and 19.44% compared to the silver Band-Aid-associated silver nanoparticles, respectively.^162^ The experimental results exhibited that the essential oil-based electrospun nanofibrous mats loaded with silver nanocomposites showed a sustained mode of silver ion release in the simulated wound system. Therefore, due to the sustained release of silver ions from the nanofibers, very low cytotoxicity was observed upon interaction with human white blood cells and human red blood cells. Thus, the fabricated EuME-AgNPs-NFs with sustained release of silver ions and efficient antibacterial activity may provide a suitable microenvironment for wound healing applications and can be applied for diabetic wounds in clinical practice.

**Fig. 8:**
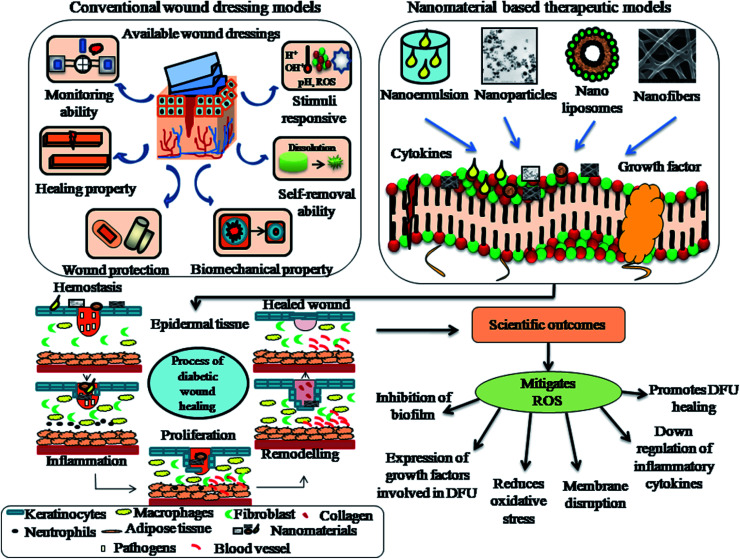
Pictorial representation of the effects and scientific outcomes of commercially available therapeutic models and nanomaterial-based therapeutic models used for the treatment of diabetic foot ulcers (DFUs). (This image has been redrawn from Dong *et al.*^204^ and Jayakumar *et al.*^205^ and the entire representation has been postulated based on the main theme of this review.)

Although electrospun nanofibrous mats have been widely employed to enhance the rate of the normal wound healing process and regeneration efficiency of damaged skin to a limited extent, the regeneration of hair follicles and functional recovery still remain unsatisfactory in the case of non-healing chronic foot ulcers. However, there are a few reports focusing on the regeneration of hair follicles in the case of DFUs. Zhang *et al.* fabricated nanofibrous mats encapsulated with bioactive anemoside B4 for enhanced wound healing efficacy in diabetic rat models. The nanofibrous wound dressing material showed multifunctional characteristics including mechanical stability, effective water absorption, and hemostatic properties with sustained mode of anemoside release behaviour. The *in vitro* results showed that the anemoside-loaded nanofibrous mats could significantly reduce the generation of reactive oxygen species (ROS) and inflammatory release of cytokines. The *in vivo* results showed that the anemoside-loaded nanofibers promoted a faster rate of wound closure, excellent angiogenesis, and regeneration of hair follicles and enhances re-epithelialization with the deposition of a collagen matrix. The newly formed epidermis and granulation tissue were found to be better structured and thicker than that in the control groups. In the latter part of the diabetic wound healing process, the regeneration of hair follicles reflected a degree of wound recovery. After 14 days, it was observed that the anemoside-loaded nanofibrous mats had generated numerous amount of hair follicles compared to that in the control groups.^163,164^ The bioactive compound anemoside B4 (ANE) was extracted from Chinese medicine *Pulsatilla* loaded in synthetic and natural polymers to fabricate electrospun nanofibrous mats, which could target various cell activities such as cell adhesion, cell proliferation, human dermal fibroblasts cell lines, and enhance healing and regeneration in the wound site. The drug-loaded nanofibers can be employed as drug delivery nanocarriers that can effectively release anti-inflammatory ingredients in the respective wound sites to enhance skin regeneration and diabetic wound healing applications. Fig. 8 describes the commonly available wound dressing models and nanomaterial-based therapeutic models for the treatment of DFUs. The effects, impact and scientific outcomes of nanomaterial-based therapeutics on the skin pathology of chronic wounds are explained in Fig. 8.

Among the different nanomaterial (nanoemulsions, nanoparticles, nanoliposomes and nanofibers)-based therapeutics used for the treatment of DFUs, nanofibrous membranes employed by the pharmaceutical sector show greater benefits with enhanced prevalence in drug delivery systems. The nanofibers utilize accessories (or) excipients to deliver therapeutic agents to the wound site with less adverse effects and great efficiency. Particularly, in DFU treatment, it is essential to focus on collagen accumulation, vascularization, re-epithelialization and physiological functions to control the deterioration process and even promote the diabetic wound healing process. In this case, nanofibers possess crucial benefits and deliver active pharmaceutical agents such as biological cells and molecules at the respective wound sites to enhance the diabetic wound healing process, absorption of exudation and exchange of water, oxygen and nutrients. In addition, nanofibers mimic the ECM and prove to enhance the process of cell attachment, proliferation and migration. Nanomaterials can be used as biological agents, drugs, nanocarriers and nanoscaffolds for the treatment of diabetic wounds. Table 6 explains the different types of nanosystems, focusing on the various modes of action and effects on *in vivo* wound healing systems.

**Table 6:** Nanosystems – effects and applications on *in vivo* wound healing systems

Mode of action	Nanosystem	*In vivo* animal models	Effects	Ref.
Intrinsic property as agents	Cerium oxide NPs	4 mm wound diameter-induced dermal wounds in male C57BL/6 mice	Accelerated proliferation and migration of fibroblasts, antioxidant nature, improved vascular endothelial cells and human keratinocytes. Complete wound closure was observed on the 13th day	165
	Levofloxacin nanoemulsion gel	Incisions of full thickness made in *S. aureus* infected streptozotocin-induced diabetic rats	Rapid epithelialization and wound contraction. Reduced biocompatibility and inflammatory cells. High induction of CD31, TGF-β and collagen synthesis intensity	166
	Fullerene derivatives	Phorbol 12-myristate 13 acetate-induced mouse wounds	Enhanced wound healing with re-epithelialization and hair follicle regeneration	167
	Zinc oxide NPs	Incisions of full-thickness wounds were made in male NCr nude mice	Fibroblast cell proliferation, adhesion of antimicrobial tissue and percentage wound closure observed on 8th day	168
Nanoscaffolds	PVA encapsulated silver nanocomposites incorporated in chitosan-agarose matrix	Excision wound models in Wistar rats	Bioeffective, biocompatible, and biodegradable with angiogenic characteristics. Tissue regeneration can be enhanced by complete fibroblast and collagen development. 95% of wound healing was observed within 9 days	169
	*Aloe vera*-encapsulated polycaprolactone nanoscaffold incorporated with fluorescent protein-labeled human umbilical cords	Excisional wounds and diabetic wound model	Secretion of superoxide dismutase, fibronectin, elastin, metalloproteinase-1, collagen I and III, keratinocyte markers along with increased expression of VEGF-A, ICAM-1 and TIMP-1. Rapid wound contraction with increased sebaceous glands and hair follicles	170
	PVA-chitosan nanofibers consisting of graphene	1 × 1 cm^2^-induced BL mouse and 2 × 2 cm^2^-induced Beveren rabbit excisional wound models	Healing was observed on days 15 and 10, respectively	171
	Chitosan/poly lactic acid nanoscaffolds	Induced diabetic rat models	Biodegradable, biocompatible, and moist-retaining scaffold. Wound healing was observed on 14th day	172
Nanocarriers	rhEGF incorporated with lipid NPs (LNPs)	Full thickness wounds of approximately 0.8 cm diameter were made in genetically modified diabetic db/db mice	Encapsulation efficiency was found higher in solid LNPs than the nanostructured LNPs. Topical administration improved wound closure. Enhanced re-epithelialization	173
	Rosmarinic acid-incorporated with chitosan nanoparticles encapsulated on carbopol 940 hydrogel	2 cm^2^ induced on excision wounds in Wistar rats	Prolonged drug release was observed up to 14 h. Complete wound contraction was observed on 21st day. Biocompatible with skin	174
	Poly(amidoamine) (PAMAM) dendrimer coated with stem cells added on E-selectin	Surgically infused with corneal and cutaneous wounds	Customized stem cell delivery and homing of healing tissues. Non-toxic mechanism. Improved neovascularization and proangiogenic effects	175
	NO- releasing hydrogel with glass composite	Full thickness wound induced on BALB/c mice	Wound closure was observed on 12th day. Intact morphological and structural characteristics, low inflammation, angiogenic effects and neutrophil infiltration levels	176

## 6. Emerging therapeutics for the treatment of DFUs

### 6.1 Commercial wound healing products

Significant innovative therapies have been developed for the treatment of DFUs such as immunomodulatory cytokines, miRNA, antimicrobial molecules, growth factor and exosomes. Antimicrobial therapies using silver or iodine are highly efficient in decreasing the microbial load present in the wound bed.^177^ The commercial wound dressings available in USA and Europe are Iodosorb and Actisorb™ Silver220. Antimicrobial peptides with wound healing properties control bacterial infection and inflammation. These physicochemical properties are desirable in topical formulations for the treatment of DFUs. Commercial medications consisting of growth factors (GFs) applied in prescribed doses and administrated over a long period have major side effects, and thus increase the cost of wound therapy.^178^ Presently, fibroblast growth factor (FGF), platelet-derived growth factor (PDGF) and epidermal growth factor (EGF) have been widely applied for GF-based wound repair. Some commercially approved products including GFs are prescribed as medications in the form of gels, ointments, solutions and creams. Recombinant human basic fibroblast growth factor FGF, recombinant human PDGF and recombinant human EGF are the available commercial formulations consisting of growth factors.^179^ Fiblast spray is a recombinant human fibroblast growth factor (FGF) product that has been commercialized in Japan. Regranex Gel is a sodium carboxymethylcellulose gel consisting of 0.01% becaplermin, which has been approved by the Food and Drug Administration (FDA).

Another important characteristic of biological-based wound dressings is their potential to interact with matrix proteins or cells in the wound bed to accelerate the wound healing process. The ECM is a combination of functional and structural proteins. These types of proteins are strongly produced by living skin cells and are triggered by the biomechanical and physiologic requirements of the skin. The three-dimensional (3D) pattern of the ECM promotes the proliferation, organization and differentiation of cells during the phase of wound healing. Porcine urinary bladder matrix, equine pericardium and porcine-derived small intestinal submucosa are the few available commercial ECM-based scaffolds.^180^ These biological products act as temporary substrates in which cells can proliferate and migrate in a controlled and well-organized manner. Although ECM scaffolds have disadvantages in the treatment of chronic wounds due to the absence of tissues and cells, the autologous mode of cellular elements has been reported in 3D scaffolds. This type of skin equivalent addresses the ECM matrix by adding collagen and provides immune living cells, which can actively synthesize and proliferate cytokines, GFs and ECM components, thus creating a suitable wound environment.^181^ Some of the commercially available skin equivalents are Dermagraft, Apligraf and Alloderm™. Dermagraft is formed by a polymeric scaffold coupled with neonatal allogeneic fibroblasts. These type of biological wound dressings offer potential for the treatment of chronic wounds given that they act strongly to improve the process of wound healing. Apligraf is an FDA product consisting of a dermal layer made up of fibroblast-seeded collagen matrix and an epidermal layer consisting of keratinocytes.^182^ MicroRNAs (miRNA) are endogenous non-coding RNA molecules involved in several biological processes including diabetic wounds. *In vivo* studies in diabetic mice have reported that miRNAs were expressed differently in skin cells and caused alternative changes in the level of expression during the process of wound healing.^183^*In vivo* upregulation of miR-335 and miR-129 enhances the percentage of wound closure through matrix metalloproteinases-9 (MMP-9) expression in diabetic models. RNA delivery techniques led to the development of wound dressings carrying anti-miRNA or stable miRNA molecules for wound healing applications. An evaluation carried out using synthetic microRNA-92a inhibitor 25 demonstrated accelerated angiogenesis and diabetic wound healing in various animal models such as normal pig and diabetic mice.^184^ Although their effects in chronic wound healing have not yet been reported, exosomal miRNAs have been obtained from human amniotic epithelial cells, mesenchymal stem cells and human blood plasma, which can be used for wound healing applications. Table 7 presents an overview on the latest commercial dressing models for DFU treatment.

**Table 7:** Commercial dressings for DFU treatment

S. no	Commercial dressing	Composition	Fabricant	Main characteristic features
1	Unite Biomatrix	Non-reconstituted collagen	Synovis Orthopedic and Woundcare, Inc	✓ allows wound closure and formation of granulation tissue
				✓ Durable and strong
				✓ Absorbs excess wound exudates, which allows dressing changes
2	Promogran matrix	ORC, collagen and silver-ORC matrix	Systagenix	✓ Permitted for compression therapy
				✓ Ideal for use
				✓ Non-irritating and non-toxic
				✓ Biodegradable gel is conformable and soft
3	Fibracol plus alginate and collagen wound dressing	Calcium alginate and collagen fibers wound	Systagenix	✓ Maintains ideal conditions for moist environment
				✓ Soft and sterile
				✓ Flexible, adherent and conformable
				✓ Collagen and alginate gel-forming properties
4	Regranex gel	PDGF-BB impregnated in sodium carboxymethyl cellulose	Healthpoint Biotherapeutics	✓ Promotes proliferation and recruitment of chemotactic cells
				✓ Easy for use
				✓ FDA approved agent with PDGF
				✓ Aids formation of granulation tissue
				✓ Enhances wound closure
5	MediHoney dressing	Consists of 95% active honey and calcium alginate	Derma Sciences Inc	✓ Natural, non-toxic, easy and safe to use
				✓ Highly osmotic
				✓ rReduces wound pH
				✓ Promotes balanced environment for wound healing
				✓ Honey possess a sustained mode of release in the wound environment
6	Sorbalgon	Calcium alginate	Hartman USA, Inc	✓ Highly absorbent
				✓ Latex-free
				✓ Forms hydrophilic gel
				✓ Maintains integrity
				✓ Easy for removal
7	Tegaderm alginate dressing	Polyurethane containing alginate	3M health care	✓ Highly absorbent wound dressing
				✓ Easily irrigated from wound bed
				✓ Forms gel-like consistency and provides moist balanced environment
				✓ Easy removal from fragile type of tissues by means of gentle irrigation
8	Biatain foam dressing	Non-adhesive polyurethane	Coloplast corp	✓ Absorbs wound exudate and protects heel
				✓ Low risk of maceration or leakage
				✓ Effective and safe
				✓ Prevents skin maceration
				✓ Beveled edges makes more comfortable for DFU patient
9	DuoDERM CGF	Polyurethane foam	ConvaTec	✓ Promotes formation of granulation tissue
				✓ Minimizes skin trauma
				✓ Promotes observation of healing process
				✓ Can be easily molded into place
10	Allevyn	Polyurethane film combined with foam consisting of 5% silver sulphadiazine	Smith & Nephew, Inc	✓ Retains, absorbs and transpires wound exudates
				✓ Provides balanced environment for faster rate of wound closure
				✓ Sustained and rapid mode of antibacterial activity
				✓ Stays up to 7 days
				✓ Reduces pain in the wound area
11	Ligasano	Polyurethane foam-honeycomb	Ligasano	✓ Manageable and economic
				✓ Stimulates blood circulation in the wound environment
				✓ Creates warm and moist environment
				✓ Absorbs wound exudates
				✓ Acts as an antiseptic and antibiotic
12	Bionect	Contains 0.2% hyaluronic acid	Dara Bioscience	✓ Reduces high-grade skin reactions
				✓ Easy and ideal for use
				✓ Decreases wound severity
13	BGC matrix	Carbohydrate beta-glucan and collagen	Molnlycke health care US, LLC	✓ Flexible, adherent and conformable
				✓ Collagen aids hemostasis
				✓ Promotes structural support
				✓ Protects tissue from contamination
				✓ Minimizes pain in the wound area
14	Collagen and Dermacol/Ag matrix dressing	Sodium alginate, ethylenediaminetetraacetic acid (EDTA), collagen, carboxymethyl cellulose and silver chloride	DermaRite Industries	✓ Maintains ideal conditions for wound healing
				✓ Easy for use
				✓ Antibacterial silver chloride prevents dressing colonization
				✓ Transforms into gel sheet upon interaction with fluid exudates
15	Aquacel dressing (hydrofiber)	Antibacterial hydrofiber consisting of carboxymethyl cellulose associated with ionic silver	ConvaTec	✓ Retains and absorbs wound exudates
				✓ Helps to decrease trauma and pain
				✓ Conforms wound surface
				✓ Used especially on diabetic wounds
				✓ Maintains a balanced environment
16	Algisite with calcium alginate wound dressing	Calcium alginate	Smith & Nephew, Inc	✓ Easy removal
				✓ Conforms to wound contours
				✓ Prevents formation of scars and enhances wound contraction and wound closure
				✓ Helps to reduce trauma
				✓ Promotes gaseous exchange
17	Kaltostat wound dressing	Calcium and sodium salts of alginic acid	ConvaTec	✓ Easy and ideal for use
				✓ Promotes wound healing
				✓ Calcium ions promote wound healing to take up a gel appearance
				✓ Facilitates micro-environment and absorbs wound exudates
18	GranuDerm	Alginate hydrocolloid associated with polyurethane	Acute care, LLC.	✓ Dirt, water and germ proof
				✓ Reduces the frequency of dressing
				✓ Prolonged wear time
				✓ Enhances the percentage of wound healing
				✓ Prohibits wound leakage
19	MANUKAhd	Polyurethane film and film containing polyacrylate polymers with ManukaMed honey	ManukaMed, Inc	✓ Permeable to fluids
				✓ Gentle upon interaction with wounds
				✓ 100% efficient medical grade
				✓ Absorbs wound exudates
20	Mepilex Ag	Polyurethane foam containing of silver sulphate	Molnlycke care	✓ Waterproof and absorbs wound exudates
				✓ Maintains moist balanced environment
				✓ Vapor-permeable

### 6.2 Bioengineered skin substitutes for DFUs

A wide variety of skin substitutes based on plasticity and composition has been used for wound healing. They can be classified into matrices that contain autologous cells, allogeneic cells and acellular matrices. The cell-containing matrices are mainly composed of keratinocytes, fibroblasts and other living cells. Acellular matrices serve as ECM scaffolds to support the migration and revascularization of fibroblasts.^185^ Apligraf/graftskin is an allogeneic cultured skin equivalent that has been isolated from neonatal foreskin. It has an epidermal layer consisting of keratinocytes and dermal layer consisting of fibroblasts. Apligraf coupled with wound care, offloading and debridement shows a progressive trend in the healing process of neuropathic ulcers. A statistically significant increase in the rate of ulcer healing was seen compared to the control group.^186^ Dermagraft is a type of fibroblast-isolated dermal substitute. It is isolated from neonatal human dermal fibroblasts that have been cultured *in vitro* using a bioabsorbable mesh. Patients treated for DFUs for more than six weeks exhibited a clinical benefit upon treatment with Dermagraft *versus* conventional therapies alone. Nearly 30.0% of the treated Dermagraft patients were healed compared to the control group.^187^

Allogeneic membranes isolated from the placenta have been used for the treatment of DFUs, which can be isolated after caesarean deliveries. The placental membrane supplies structural collagen, GFs and cytokines, which are involved in the process of tissue repair. The conventionally available placental membrane allografts include Grafix, an amniotic product, and Epifix, a chorion membrane allograft that delivers ECM proteins, chemokines, non-viable cells, active GFs and cytokines, which showed a higher percentage of ulcer healing compared to Apligraf and other standard wound care products.^188^ In a clinical trial involving 110 patients, a significant percentage of ulcer healing was observed with the use of an allograft. Laserskin, Hyalograft and TransCell are autologous cell-containing matrices, which were isolated from the skin biopsies of patients. TransCell has been developed as a carrier surface for autologous keratinocyte transfer to promote the wound healing process. It is regarded as a medical grade polymer, which consists of 20% carboxylic acid, allowing keratinocytes to proliferate and migrate. The isolated keratinocytes are thawed and transferred to TransCell 48 h prior to wound administration.^189^ A few studies have reported that the healing of neuropathic ulcers using autologous keratinocytes has been delivered through transfer discs and cell-free discs. A global reduction in the area of chronic ulcers has been observed in response to the active treatment of skin substitutes.^190^ Integra Dermal Regeneration Template, GraftJacket, Bilayer Matrix Wound Dressing, Dermacell and OASIS Wound Matrix are considered acellular matrices for the treatment of DFUs. GraftJacket is an allograft isolated from human skin, which acts as a scaffold for vascular and cellular growth in wounds. A randomized multicentre trial evaluated the efficiency of GraftJacket compared to hydrogel/alginate dressings in the healing of chronic wounds.^191^ The results showed that 69.6% of the patients belonging to the GraftJacket group were healed at 5.7 ± 3.5 weeks compared to the control group, which healed at an average of 6.8 ± 3.3 weeks. Two other important matrix-based commercial products are Integra and Dermacell. Integra is mainly composed of chondroitin sulphate and bovine collagen with a covering of silicone membrane.^192^ Integra can be used to treat DFUs in a two-phase trial containing nearly 307 patients. After treatment with a 16 week follow up, the individuals treated with the Integra graft had an increasingly higher rate of wound closure compared with the control group. The week-wise reduction in the wound was approximately 7.2% compared with the control group, which was only 4.8%.^82^ A composite paste made up of acellular Dermal Matrix was developed for the treatment of ulcers, which showed 83% reduction in the treatment group compared to the control group, which showed only 41%. No complications after application of the formulated composite paste have been reported, making it more attractive than when applied as a film.^193^ Treatments such as Dermagraft, GraftJacket, 3D Kaloderm, Apligraf, Epifix, Hyalograft and OrCel are a few effective skin grafts and tissue replacements for the treatment of DFUs. These tissue replacements and skin grafts compared to standard wound care show enhanced diabetic wound healing applications.

### 6.3 Nanomaterials – innovative therapies

#### 6.3.1 Nanomaterials for antibacterial (hyperthermia) treatment

One of most important nanomaterial-based innovative strategies is hyperthermia treatment to prevent or cure bacterial infections. The basic idea is to destroy the bacterial cell wall by stimulating irreversible hypothermic energy with an applied energy source such as NIR light or alternating magnetic field (AMF).^194^ Primarily, nanomaterials absorb external energy and produce heat, which increases the surface temperature and damages bacterial cells. It is remarkable that bacteria are disrupted at 55 °C due to the degradation of their heat-shock proteins.^195^ The photothermal effect demonstrates a high influence on the thermal conversion efficiency due to the irradiation of NIR light. In addition, NIR light in the wavelength range of 700–1400 nm penetrates mammalian cells, which causes damage to normal living cells. Thus, photothermal therapy (PTT) is considered an efficient, safe and innovative approach to deal with wound healing aspects or microbial infections. Among the available nanomaterials, iron oxide, carbon nanotubes (CNTs), Au, graphene and BP are suitable photothermal agents due to their intense optical property. A polydopamine-based hydroxyapatite (PDA@Au-Hap) nanocomposite material helped to enhance the antibacterial activity at approximately 45 °C and prevented damage to normal tissues. It played an important role in granulation tissue formation with the synthesis of fibroblasts and collagen and diabetic wound healing applications.^196^ The prepared nanomaterial exhibited a faster rate of wound healing compared to the control group. Chiang *et al.* developed a hybrid microsphere with core–shell based polypyrrole nanoparticles impregnated with vancomycin, where polypyrrole nanoparticles were considered as a photothermal agent. The polypyrrole-based nanoparticle combination synergistically eradicated a greater amount of bacteria in wound abscesses than the total of individual treatment approaches.^197^

Another important nanomaterial-based approach is AMF-associated magnetic hyperthermia. In this particular approach, the heating capacity of magnetic nanoparticles was utilized with the influence of high-frequency AMF (>100 kHz). The magnetic nanoparticles absorb electromagnetic radiation and transmit localized heat on the surface of the nanomaterial, which has the capacity to kill bacterial pathogens.^198^ A synergistically combined approach using d-amino acids with hyperthermia successfully dispersed and inhibited the formation of biofilms. Primarily, d-amino acids disrupt the exopolysaccharides of biofilms, following AMF exposure. This particular approach eradicated biofilms of *Staphylococcus aureus* with less toxicity to mammalian cells.^199^ Kim *et al.* applied AMF-associated magnetic hyperthermia to damage the biofilms generated by *Staphylococcus aureus* in studies conducted in *in vitro* and *in vivo* models.^200^ A critical structural design is essential for safe wound healing applications.

#### 6.3.2 Nanomaterials – gene nanotherapy

Gene-stimulated matrix-based therapy has been used recently for the regeneration of bone, skin and cartilage. DNA stability is the major advantage of gene therapy for wound healing applications. Current techniques such as gene gun transfer, direct injection and electroporation deliver DNA to the wound sites that need injections but hindered by short-term and inconsistent gene expressions.^201^ This can be resolved scientifically using nanomaterials such as electrospun mats/meshes used as matrices for wound dressing materials and gene encapsulation. Kobsa *et al.*^202^ reported that nanofibrous scaffolds of poly-ε-caprolactone (PCL) and poly(lactic acid) (PLA) can be used for the localized delivery of plasmid DNA impregnating keratinocyte growth factor, which is more efficient in treating cutaneous wounds. Tong *et al.* guided single-stranded DNA (ss DNA) with AgNPs on GO and reported excellent antimicrobial activity, which cured wound infections caused by *Staphylococcus aureus*.^203^ However, gene-based approaches for diabetic wound healing applications are still required for effective epithelialization and re-vascularization. Many commercially available wound dressings such as hydrogels, bandages, films and foams help the diabetic wound healing process but not as efficiently as expected.^204^ However, nanomaterial-based therapeutic models possess a longer and extended shelf life, high antimicrobial efficacy, mitigation of ROS and oxidative stress, sustained and controlled mode of drug release, great potential against resistant strains and efficient re-epithelialization with vascularization ability.^205^

## 7. Conclusions and future perspectives

Despite the numerous advances in the therapies for diabetic wound healing, the difficult task in skin regeneration is the generation of a tissue containing hair follicles, microvessels and sweat glands. Only a few studies were reported on the regeneration of hair follicles and efficiency of skin recovery in the case of non-healing chronic ulcers. This review is different from other reviews in that it reviews the status and prevalence of diabetes and diabetic wound amputations in India and various types of nanomaterial-based approaches (such as nanoparticles, nanofibers, nanoemulsions and nanoliposomes) for the treatment of diabetic wound healing, which were discussed. In addition, emerging therapeutics in terms of commercial wound products, bioengineered skin substitutes and nanomaterial-based innovative strategies such as antibacterial treatment and gene therapy for the treatment of DFUs were studied. The nanomaterial-based approaches for diabetic wound healing process have been proven to be much more efficient than the conventional wound therapies, which basically depend on the type of wound dressing model. Nanomaterials help to alter two or more phases of the wound healing process due to their anti-inflammatory, antibacterial and anti-proliferative properties. However, although nanomaterials play an important role in resolving major issues in wound healing therapy, there are still some important concerns to be addressed for their diabetic wound healing applications. Future measures on nanomaterial-based innovative strategies should focus on targeting sites for effective diabetic wound healing applications depending on the nanomaterial used. Another major problem is that there is very little knowledge on nanomaterial-mediated diabetic wound healing applications, and also the toxicity of nanomaterials is still one of the principal concerns because it has an adverse effect on human cells. Nanomaterials incorporated with stem cells, growth factors, essential oils and other organic components should be fabricated to treat multi-drug resistant bacteria (Gram-positive and Gram-negative) and infections. Therefore, scientists should focus on developing biodegradable and biocompatible nanomaterials that have the potential to reduce the threat of diabetic foot ulcers and reduce the number of limb amputations and prolonged hospitalizations in India.

## Conflicts of interest

All authors declare no professional or personal conflicts of interest.

## Supplementary Material
